# Integrating Immunology and Microfluidics for Single Immune Cell Analysis

**DOI:** 10.3389/fimmu.2018.02373

**Published:** 2018-10-16

**Authors:** Nidhi Sinha, Nikita Subedi, Jurjen Tel

**Affiliations:** ^1^Laboratory of Immunoengineering, Department of Biomedical Engineering, Eindhoven University of Technology, Eindhoven, Netherlands; ^2^Institute for Complex Molecular Systems, Eindhoven University of Technology, Eindhoven, Netherlands

**Keywords:** immunoengineering, microfluidics, single-cell analysis, cellular heterogeneity, cellular communication

## Abstract

The field of immunoengineering aims to develop novel therapies and modern vaccines to manipulate and modulate the immune system and applies innovative technologies toward improved understanding of the immune system in health and disease. Microfluidics has proven to be an excellent technology for analytics in biology and chemistry. From simple microsystem chips to complex microfluidic designs, these platforms have witnessed an immense growth over the last decades with frequent emergence of new designs. Microfluidics provides a highly robust and precise tool which led to its widespread application in single-cell analysis of immune cells. Single-cell analysis allows scientists to account for the heterogeneous behavior of immune cells which often gets overshadowed when conventional bulk study methods are used. Application of single-cell analysis using microfluidics has facilitated the identification of several novel functional immune cell subsets, quantification of signaling molecules, and understanding of cellular communication and signaling pathways. Single-cell analysis research in combination with microfluidics has paved the way for the development of novel therapies, point-of-care diagnostics, and even more complex microfluidic platforms that aid in creating *in vitro* cellular microenvironments for applications in drug and toxicity screening. In this review, we provide a comprehensive overview on the integration of microsystems and microfluidics with immunology and focus on different designs developed to decode single immune cell behavior and cellular communication. We have categorized the microfluidic designs in three specific categories: microfluidic chips with cell traps, valve-based microfluidics, and droplet microfluidics that have facilitated the ongoing research in the field of immunology at single-cell level.

## Introduction: immunoengineering

The human immune system recognizes myriads of environmental triggers and is highly flexible in generating a variety of signaling responses over time (1, 2). Several types of cells collaborate with antibodies and cytokines to generate an appropriate immune response (3). The spatial organization and migration of cells within tissues as well as the dynamic nature of cellular communication enhances the complexity of our immune system and determines the type of response (4–7). The nature and magnitude of an immune response is dependent on dynamic molecular and cellular interactions where well-orchestrated cellular communication is the key factor to maintain it (8). The question arises whether all immune cells fight all pathogens and tumors similarly in order to leverage this broad flexibility and diversity. Even though there are multiple subsets of immune cells, with each subset responding to specific stimuli, responses are often initiated by individual cells within each subset and communicated in order to establish a more complex population level response (9). Stochastic expression of genes (influenced by a cellular microenvironment) or pre-defined molecular drivers (as in case of B-cell and T-cell receptors) are driving factors behind heterogeneity in the human immune system (9). Numerous studies over the last decades established that heterogeneity is a trademark of the human immune system (10, 11). The identification of heterogeneity requires systems beyond conventional biological methods like ELISA, western blot, and others that do not allow the required spatial and temporal manipulation of biological cells (12–14). Technologies such as microsystems and microfluidics, have allowed scientists to study the individual behavior of immune cells, identify signaling pathways, map distinct immune cell subsets, quantify secreted molecules and characterize the immune response under varied conditions (15–17). Research on immune cells with technology integration has contributed toward innovative immunotherapy-based treatment modalities with lower treatment-related toxicity and side effects (18–20). They provide better alternatives to more conventional treatment modalities e.g., chemotherapy, radiotherapy or targeted therapy. At the same time, microfluidic based models have assisted in the development of novel therapies, discovery of new drugs, and monitoring the clinical efficacy of new treatments (21–23). Microfluidic devices are also used for the isolation of circulating tumor cells from clinical samples for diagnosis, prognosis, and creation of patient-derived tumor models with the aim to develop and test personalized medicine (24–27). Furthermore, integration of assays, microarrays, and several sensor technologies has led to the development of several point-of-care devices for the early diagnosis of cancer by identification of cancer biomarkers (28, 29).

**Figure 1:**
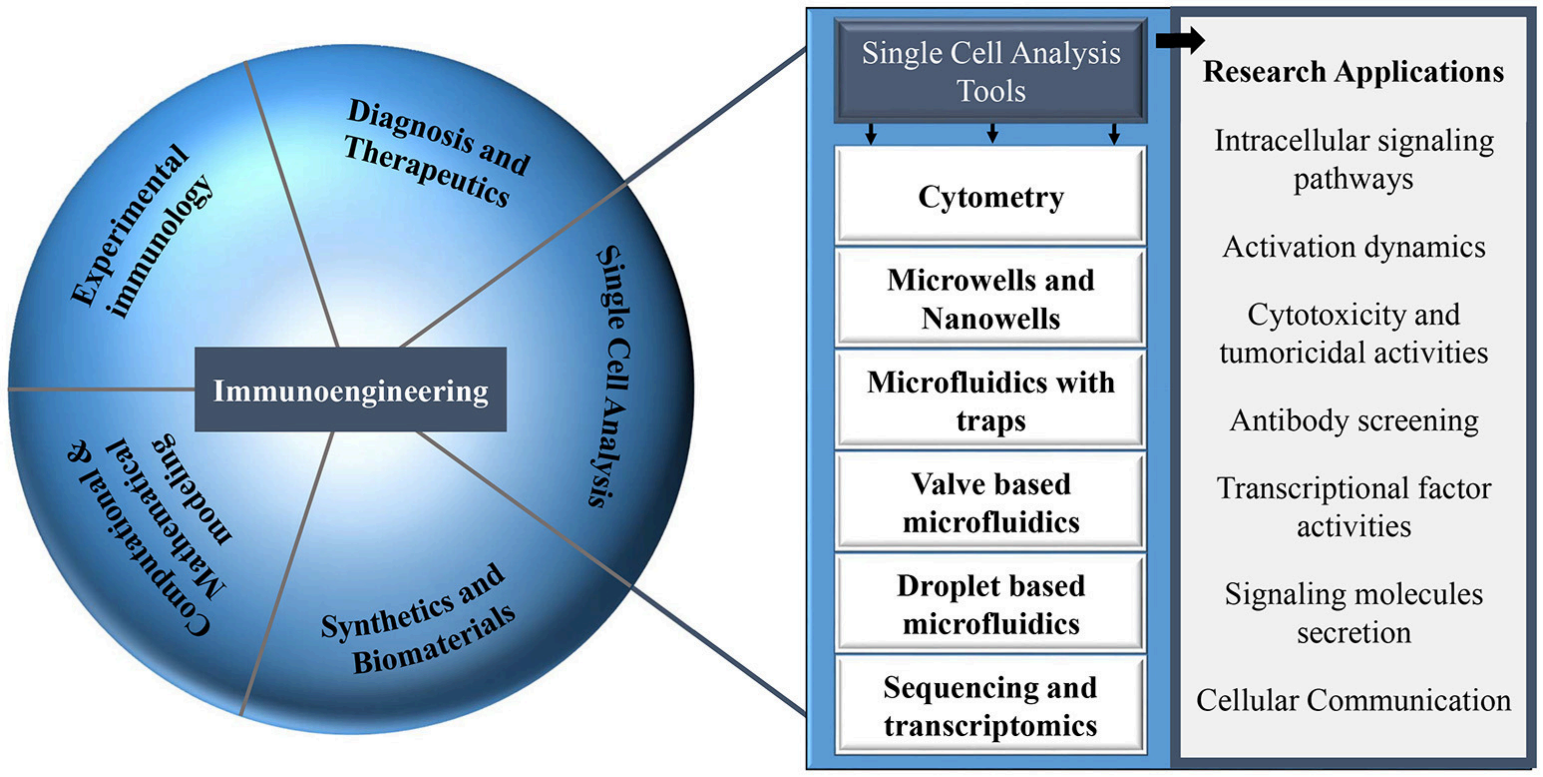
Different research areas in the field of immunology that can be explored with multiple single-cell analysis tools.

The majority of this type of research can be coined as immunoengineering. The term immunoengineering has been used since the seventies and covers several aspects in the field of immunology (30). Immunoengineering is an interdisciplinary and vast field of research comprising engineering methods and approaches that allow the modulation of the immune system and its responses: biomaterials, tissue engineering, protein engineering, synthetic biology, and drug delivery systems [Figure 1; (31–34)]. Immunoengineering includes the application of systems immunology to replicate complex immune microenvironment, *in vitro*, that aims to enhance our understanding of the human immune system for development of immunotherapy, the modulation of the human immune cells to boost their response against cancer (35–37). Recent and most noteworthy examples of major discoveries within the field of immunoengineering, specially immunotherapy, are the development of chimeric antigen receptor T-cells and artificial antigen presenting cell systems (38–40). Moreover, immunoengineering also involves mathematical models that describe the functioning of the immune system, technologies to monitor and track the migration of immune cells and engineering tools to understand immune cell function at the systemic level in health and disease (41–43). The field of immunoengineering can, and has been described by various definitions, e.g., for the current issue in Frontiers “Application of systems immunology to engineering the tumor immunological microenvironment, aiming at predicting lymphocyte receptor's recognition patterns. Building sophisticated mimetic *in vitro* models, for instance by means of optical and magnetic tweezers to develop novel immuno-oncotherapeutics paving the way toward personalized and predictive medicine.” The Center for Immunoengineering at Georgia Tech University defined this field as follows: “The field that applies engineering tools and principles to quantitatively study the immune system in health and disease, and to develop new therapies or improve existing therapies by precisely controlling and modulating a patient's immune response.” The field of immunoengineering has been described in excellent reviews with a focus on engineering approaches to augment immunotherapy (44–48). In this review article, we highlight one aspect of immunoengineering and we particularly discuss various microfluidic and microsystems and focus on their advantages over conventional methods especially for decoding heterogeneous immune cell behavior and cellular interactions.

### Single-cell technology

Immune cells, characterized by their heterogeneity, tend to differ in their behavior when in different societal contexts ranging from the single cell to the population level. Experiments performed at the population level average out the behavior of all the individual cells (49). Hence, bulk studies fail to provide a coherent understanding of the immune system by masking the phenotype, expressed genes, proteins or metabolites at single-cell level, and cellular communication between single immune cells (49, 50). The advent of single-cell technologies and the subsequent possibility to study the behavior of individual immune cells has uncovered various biological functions that were previously not detectable with bulk studies (51–53). For instance, Shalek et al. demonstrated the importance of paracrine communication for generation of immune response using single-cell analysis (54). Single-cell analysis enabled the investigation of maturation, activation, and signaling pathways of individual immune cells triggered by various environmental factors as well as intercellular communication between different immune cells (43, 55, 56). Additionally, this approach identified new immune cell subsets (57, 58). For instance, single cell transcriptomics, introduced a paradigm shift in the CD4+ T helper field and enabled the identification of multiple functionally distinct T helper cell subsets in addition to the two well-established subsets, Th1 and Th2 (59–62).

Single-cell technology requires isolation of individual cells from a population for multiple data extraction from each isolated cell in order to gain information on the genotype, phenotype, lineage, protein secretion, proliferation, activation, maturation, cytolytic activity, and intercellular communication (63). Single-cell analysis tools are currently investigated by various research groups worldwide and hold great promise in providing a comprehensive understanding of our immune system. After the isolation of individual immune cells, multiple experimental operations for DNA sequencing, RNA and protein expression profiling can be implemented to map the lineage and identify subsets of immune cells (12, 64, 65).

Amongst immunologists, flow and mass cytometry are well-established, high-throughput, and high-content single-cell analysis tools (66–68). Flow cytometers measure fluorescently labeled cells and mass cytometers use transition element isotopes for mapping the functional heterogeneity and phenotypes of different immune cells by quantification of multiple cytokines, chemokines, and surface protein markers of the individual cells (69, 70). One of the benefits of cytometry over other conventional methods is its potential to provide high-throughput analysis of thousands of single-cells and measure multiple parameters in a given time frame (71, 72). Further, with recent advancements, mass cytometry can acquire samples using laser ablation to improve the resolution of this technology and is known as imaging mass cytomtery (73). Although cytometers are a powerful tool for single-cell analysis, spectral overlap and limited availability of antibodies labeled with isotopes for flow and mass cytometers, respectively are some of the drawbacks of this technology (13). Even though spectral overlap in conventional cytometry can be effectively mitigated by careful panel design, cytometers are still predominantly an end-point measurement tool that can only provide a snapshot in time and quantify static markers on cells to provide information on immune cell heterogeneity.

### Microsystems for single-cell analysis

The requirement for miniaturization of technological platforms has driven the development of several technologies such as microtiter plates (74). However, with microtiter plates, reaction volumes of immunological experiments have only been reduced from milliliters to microlitres. The problem of evaporation and capillary action in microtiter plate technology has hampered its further miniaturization (75). While microtiter plates cannot be further scaled down, the field of microsystems and microfluidics has played a key role in miniaturization to propel interdisciplinary research on single-cell analysis of immune cells.

**Figure 2:**
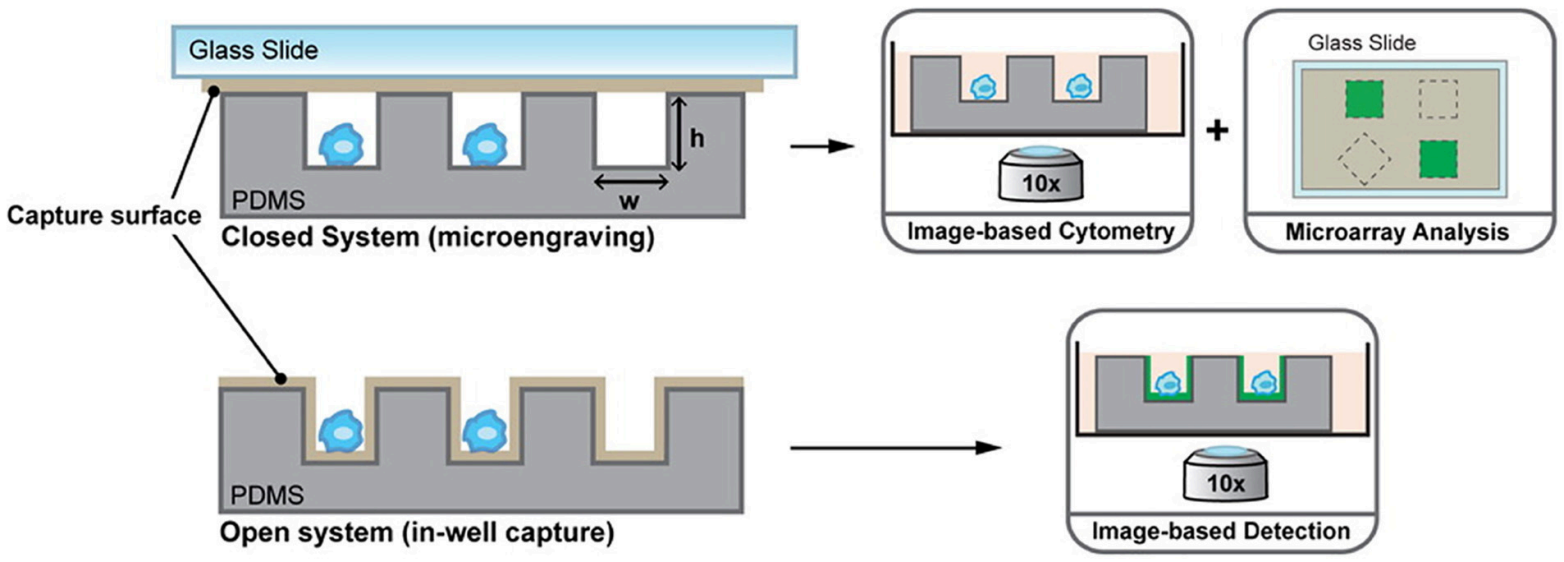
Nanowell format array, developed at the Love laboratory, to understand activation dynamics of immune cells, probe cellular interactions, and quantify cytokine secretion. Figure adapted from Torres et al. (77).

Driven by the idea of scaling down, nanowells, in combination with microengraving, is a microsystems tool that was developed in the Love laboratory for single-cell analysis (76). Nanowells, made from polydimethylsiloxane (PDMS), contain features that have volumes in the order of nanolitres. When cells, from a bulk solution, are dispensed on the platform, individual cells settle down in each nanowell by gravity. Once the cells are isolated and activated, secreted molecules from the cells can be captured on functionalised glass slides (microengraving) or on the surface of nanowells and quantified by imaging cytometry or microscopy [Figure 2; (76, 77)]. Nanowell-based platforms with imaging cytometry and microarray analysis have been used for quantification of cytokines secreted by T-cells and observation of T-cell proliferation when activated with an array of ligands (CD80, major histocompatibility complex class II/peptide and intercellular adhesion molecule-1) or anti-CD3/CD28 (78). Nanowells can also be used for cell-pairing to study intercellular immune cell interactions and to monitor cytotoxic effector functions of immune cells (79). In 2017, An et al. presented their work on natural killer (NK) cells (80). In their study, they dynamically profiled the secretion of interferon gamma (IFN-γ) from single NK cells to map the phenotypic behavior of these cells based on their cytokine secretion pattern. Using this system, they showed that CD56^dim^CD16+ NK cells, when activated with phorbol 12-myristate 13-acetate and ionomycin, immediately secrete IFN-γ and that the secretion rate and amount of IFN-γ from these cells is dependent on the donor.

To study complex biological systems, it is essential that technology platforms replicate the cellular microenvironment accurately and also provide precise control over it. Microfluidic devices have been instrumental in providing automated platforms to perform all the essential functions for single-cell analysis of immune cells, on-chip. In the forthcoming sections, we discuss the contribution of microfluidics to the field of single-cell analysis, focused on immune cells, along with its advantages and disadvantages.

### Microfluidics for single-cell analysis

Over the last decade, microfluidics has made significant contributions to the field of single-cell analysis. This method allows cells to be monitored dynamically with a high degree of control over the cellular microenvironment (81, 82). These approaches have offered new information by creation of innovative conditions that are limited in conventional bulk methods. Microfluidic systems are being developed for applications in several areas such as protein purification and PCR on a drastically decreased scale (83, 84). Microfluidic chips are capable of accurately replicating *in vivo* biological environments and allow high-throughput analysis of cells (85). Microfluidics allows precise automation and control of analytical functions as well as manipulation of cells and their microenvironments with high resolution in both space and time (86, 87). With microfluidics, scientists can implement techniques and protocols for single-cell analysis through DNA sequencing, RNA expression, and protein quantification for understanding the mechanism of cell activation, proliferation, protein expression, motility and morphology, secretion, and cellular communication (88–91).

The ability to rapidly fabricate microfluidic devices in PDMS by soft lithography has greatly stimulated the development of several microfluidic designs (92). Besides being inexpensive, PDMS is biocompatible and permeable to gases, two properties that are a necessary for replication of artificial cellular microenvironments *in vitro* (93, 94). The flexibility of PDMS allows easy integration of membrane valves and pumps on more complex microfluidic designs to create an intricate network of microchannels wherein protocols can be realized in full automation with the help of programming software (95).

Microfluidic chip designs can be broadly classified in three categories: microfluidics with passive traps, valve-based microfluidics, and droplet microfluidics. With pros and cons of each design, in the field of immunology, microfluidics finds its applications in understanding immune cell behavior at single-cell level.

#### Microfluidic chips with cell traps

Microfluidic chips with cell traps have been designed by multiple laboratories for single-cell studies (96, 97). In 2006, Di Carlo et al. designed a microfluidic chip with an array of hydrodynamic cell traps for analysis of enzyme kinetics in three different types of cells (98). Later in 2009, Faley et al. presented their design of a microfluidic chip with multiple traps that was used to study signaling dynamics of isolated, individual, hematopoietic stem cells (99). Besides these studies, several groups have used hydrodynamic cell trap arrays for multiple applications in biology and chemistry (100–102). Of all these groups, the Voldman laboratory has extensively used a modified version of the hydrodynamic cell trap design for specifically studying immune cell interactions at single-cell level (103–106).

**Figure 3:**
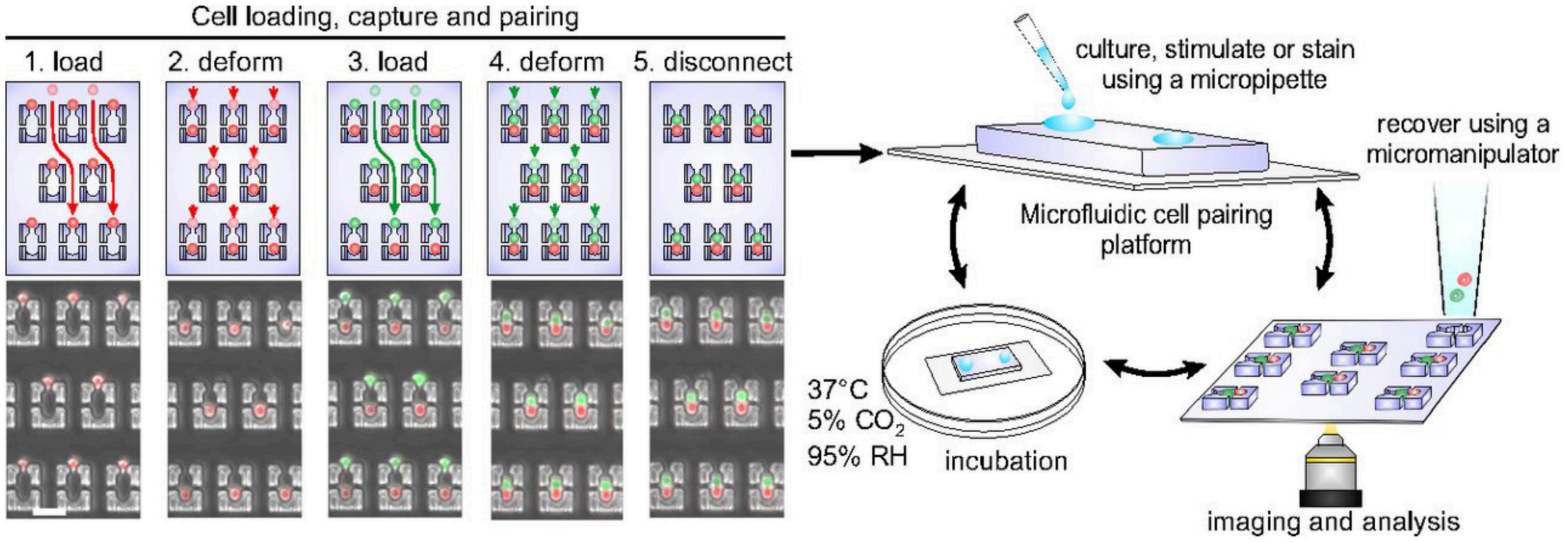
Microfluidic chip, developed in the Voldman laboratory, with hydrodynamic based pillar-like traps. There are multiple variations of this design that allow different types of cells to be loaded and paired to monitor cellular interactions of different kinds. Figure adapted from Dura et al. (105).

Cellular interactions play a vital role in establishing complex immune responses that originate from individual immune cells or immune cell subsets (107). Interactions between immune cells, if hampered, can cause several diseases (108). Hoehl et al. designed a single-layer microfluidic chip for parallel analysis of immune cell interactions at single-cell level (109). They used this design, integrated with weir like U-shaped traps, to pair two different cell types using a three-step loading protocol to obtain cell pairing efficiencies of more than fifty percent. The device was used for pairing murine T-cells with B cells to investigate the activation dynamics of T-cells. They demonstrated the presence of functional heterogeneity in the activation dynamics of OT-1 T-cells. Since OT-1 T-cell are reactive against ovalbumin, it is expected that all the T-cells will show similar activation profile when presented with the antigen. However, within the population of these cells, a variation in response was still observed even though all the cells bear the same identical T-cell receptor (TCR). Later, Dura et al. used this design to characterize the activation dynamics of CD8 T cells (OT-1 and TRP1) with different TCR affinities when paired with antigen presenting cells (104). This study showed that the variations in TCR affinity influences the secretion of cytokines by T-cells. The production of IFN-γ is strong for both low and high TCR affinity whereas the production of interleukin-2 (IL-2) reduces with reduction in the affinity. Dura et al. also used this chip to monitor cytotoxic effector functions of immune cells [Figure 3; (105)]. For this study, they modified the design of the cell traps to capture and pair NK92MI and K562 cells with high cell-pairing efficiency. The cytotoxic activity of NK cells was monitored by measuring the Ca^2+^ signaling for a day and further, the production of IFN-γ in NK cells, when activated with IL-2 and IL-18, was also quantified. Thirty-five percent of the cells showed cytotoxicity and 60% of the cell population produced IFN-γ over time, demonstrating cellular heterogeneity (105).

Besides applications in immune cell interactions, this design was also used for implementation of cell pairing and fusion protocols. In 2009, Skelley et al. paired fibroblasts, mouse embryonic stem cells, and myeloma cells, on-chip, to implement a more efficient electrical and chemical fusion protocol in comparison to the standard procedures (103). Further in 2014, Dura et al. modified the trap design to implement hydrodynamic and deformation based pairing and biologically, chemically, and electrically stimulated fusion of cells, on-chip (106). Kimmerling et al. in 2016 also used the principles of hydrodynamic trapping to design a microfluidic chip with an array of traps for the isolation of single murine leukemia cells, L1210, and primary CD8+ T-cells on chip (64). The isolated cells were cultured on-chip and, after proliferation, these cells were released from the chip for investigating their transcriptomic profiles.

Microfluidic chips with cell traps have made significant contributions and have opened opportunities to investigate different immune cells to attain improved insights into cell-cell communication by allowing deterministic one to one cell pairing which is not easily attainable with other technologies.

#### Valve-based microfluidics: microfluidic large-scale integration technology

Microfluidics large-scale integration (mLSI) is the integration of hundreds to thousands of pneumatic membrane valves, arrayed as fluidic multiplexers, on a microfluidic chip (95). Fluidic multiplexers, analogous to electronic multiplexers, allow complex manipulation of fluids with very small number of inputs. Valve-based microfluidic devices are made by aligning two separately cured PDMS layers with channels in such a way that the pneumatic membrane valves are formed when the channels in the two layers intersect each other orthogonally. These pneumatic membrane valves are “push down” when the control layer is on top of the flow layer and are “push up” when the alignment is reversed. The channels in the control layer are pressure driven and responsible for the actuation of pneumatic membrane valves. The multiplexer on the chip helps in automation and parallelisation of experimental workflows and the pneumatic membrane valves on the chip help in fluid routing, metering, and control (110). mLSI integrated microfluidic devices are controlled using external units and can be programmed to operate for days, allowing them to monitor immune cell activity longitudinally (111).

mLSI devices are versatile and have been adapted for highly complex biological applications including cellular studies and genomic analysis (112–115). mLSI technology has been extensively used by the Quake lab for isolation of mRNA, synthesis of cDNA, and purification of DNA using fully-automated microfluidic chips (116, 117). Furthermore, they used a similar architecture for the ligation and transformation of genes with sample volumes of the order of a few nanolitres (118). In 2014, Ketterer et al. used a highly multiplexed microfluidic chip to develop a sensory system for quantification of metabolites from cellular samples (119). Blazek et al., from the same laboratory, implemented a proximity ligation assay on a fully automated microfluidic chip for analysis of phosphorylation kinetics in cells with high-throughput and parallel analysis (120, 121). The Maerkl lab also focuses on implementation of highly multiplexed and automated microfluidic designs for characterization and quantification of transcriptional regulatory network and synthesis of genes and genome on microfluidic chips (122–124).

The generation of an immune response relies on environmental cues that are sensed by immune cells in their microenvironment (125). Signals received by cells within their microenvironment are not always continuous but often dynamic and can vary both in time, intensity, and concentration (126). More specifically, environmental cues can have variable amplitude, time of exposure, concentration gradient or can have pulsatile or sinusoidal variations (126). Among these variations, pulsatile modulation holds physiological relevance as several biological cues in our body such as hormones or cytokines are released with temporal variation that in turn affects the mode of action, downstream, of signaling molecules (127). In the human immune system pulsatile bursts of environmental signals dictate cellular heterogeneity and regulate the fate of transcription factors to influence the activation of genes and determine the phenotypic response of immune cells (126). Programmable mLSI based microfluidic devices make it possible to deliver pulsatile bursts of input stimuli to immune cells in a highly controlled fashion (128, 129). mLSI chips are capable of accurately mimicking the cellular microenvironment along with a reduction in extrinsic noise or cell-cell variability to generate synchronized immune cell responses at single-cell level.

**Figure 4:**
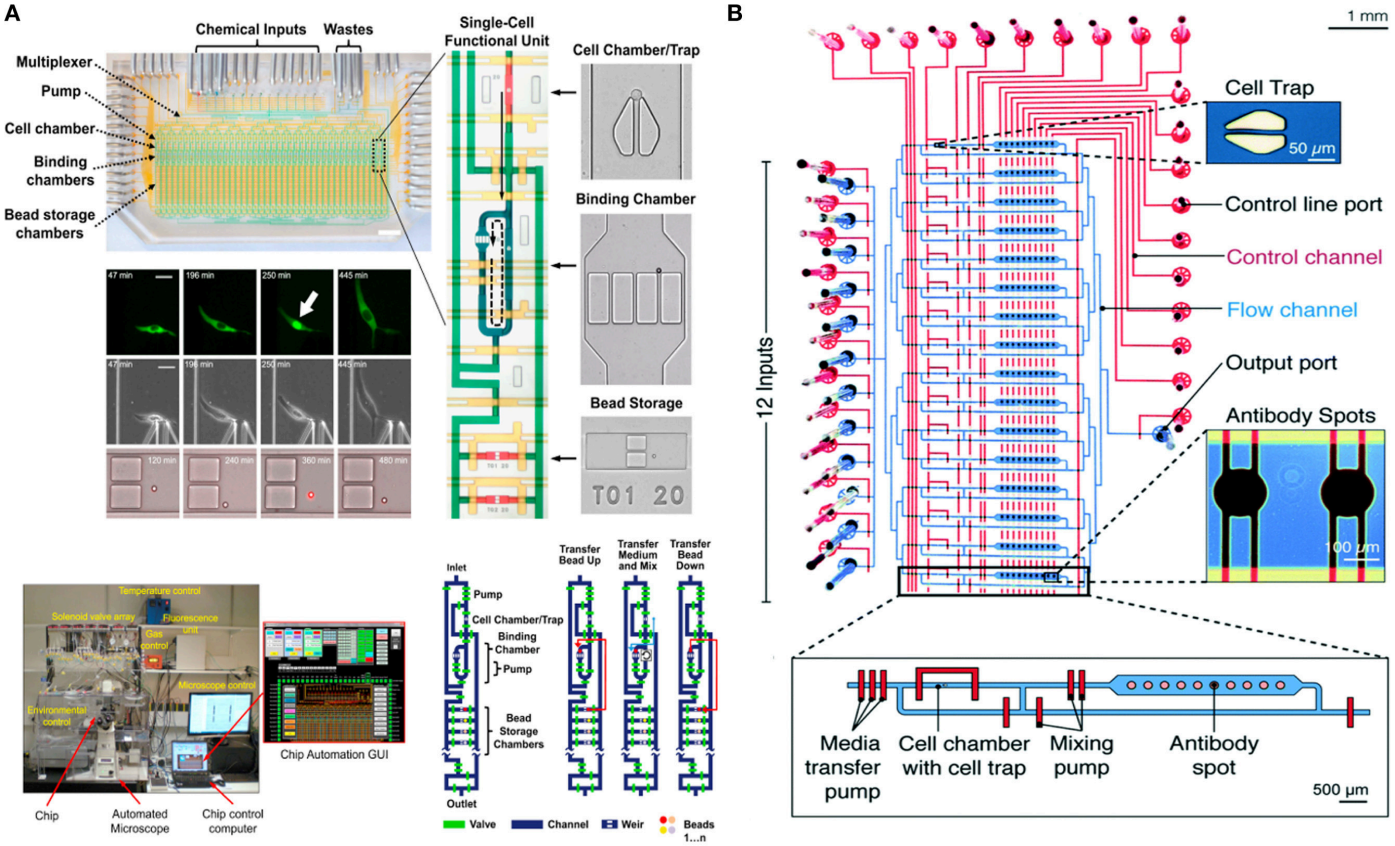
Microfluidic large-scale integration (mLSI). Microfluidic chips integrated with mLSI technology designed for monitoring transcription factor activities and quantification of secreted proteins developed in the Tay laboratory. mLSI chips allow the replication of dynamic immune system microenvironment to provide better insights to immune cell behavior**. (A)** Adapted from Junkin et al. (128). **(B)** Adapted from Kaestli et al. (129). Reproduced with permission from The Royal Society of Chemistry.

In 2016, the Tay lab designed a fully-automated microfluidic device for studying the signaling dynamics of nuclear factor-κB (NF-κB) in macrophages [Figure 4A; (128)]. In their design they precisely replicated the dynamics of the immune cell microenvironment in a highly controlled manner and complete automation at single-cell level (128). NF-κB is an important transcription factor that is responsible for production of cytokines and survival of immune cells (130). The activation and deactivation of NF-κB shows oscillatory behavior and is popularly studied using microfluidics by providing immune cells with variable input stimuli (131–133). Junkin et al. used an mLSI based microfluidic system, which was integrated with a bead-based immunoassay, to investigate the transcription factor activity and quantify cytokine secretion in macrophages when stimulated with time variable inflammatory signals from the cellular microenvironment (128). The design comprised of 40, individually addressable, cell isolation chambers in which single immune cells were trapped using pillar like structures and each cell chamber was associated with its individual immunoassay unit. For the first time, this study showed the heterogeneous secretion profile of tumor necrosis factor (TNF) when single macrophages are simulated with dynamically variable input stimuli and that there is no correlation between the production of TNF and activation of NF-κB. Very recently an immunoassay was patterned on the microfluidic chip using a modified version of the mechanically induced trapping of molecular interaction (MITOMI) method for trapping antibodies to quantify TNF secretion [Figure 4B; (129, 134)]. Since the microfluidic device is based on the mLSI technology, it was possible to completely automate the experimental workflow including the patterning of the surface for an immunoassay. In this work, they were able to stimulate cells with dynamically variable signaling molecules in a highly-precise and controlled manner as well as to monitor the activation of NF-κB in real-time using automated microscopy (129). Earlier, Frank et al. presented a microfluidic device that was used to co-culture macrophages and fibroblasts on-chip (135). This co-culture platform enabled the interaction of single immune cells with populations of cells. They used the automated device to provide dynamic inputs of lipopolysaccharide to single macrophages and monitor the signal transmission of TNF, upon activation of NF-κB, from single macrophages to a population of fibroblasts to replicate the initiation of the immune response. The experimental results of this work showed that an activated macrophage can spatiotemporally control the activation of NF-κB in fibroblasts to demonstrate that inflammation in tissues is regulated by the dynamics of gene expression (135).

mLSI technology allows automation of functional steps, on-chip, giving researchers the freedom to implement multiple experimental functions that are required for single-cell analysis. The aforementioned examples demonstrate that this technology has made valuable contributions to accurately replicate the dynamics of the cellular microenvironment with high precision and control. These designs can be further implemented to dynamically investigate the behavior of different immune cells at single-cell level.

#### Droplet-based microfluidics

The idea to perform biological analysis in water-in-oil droplets was first published in the 1950s by Nossal and Lederberg (136). Since then droplet microfluidics has continued to fuel a growing body of research leading to multiple applications in fields of biology and chemistry (137–139). Droplet microfluidics has been widely implemented for high-throughput screening of biological and chemical reactions, single-cell analysis, genomics, and transcriptomics (140–144). It also finds applications in molecular detection, imaging, drug delivery, antibody screening, toxicity screening, and diagnostics (145–151). On a microfluidic chip, using two immiscible liquids, droplets, in one liquid phase, are generated in another liquid phase by breaking off either at a T-junction or flow-focusing junction (152, 153). In such a setup, passive generation of droplets relies on drag forces and viscous dissipation (154). Variations in channel geometries help to pair, trap, merge, mix, release, and split droplets (155). Pneumatic membrane valves, electrical forces, optical manipulations and acoustic waves are other alternatives for active production of droplets on microfluidic chips (156–159).

Droplet-based microfluidic platforms provide scientists with the ability to investigate immune cell behavior in complete isolation by creating a noise-free and controllable cellular microenvironment (160). Specifically, it allows to map immune cell subsets, quantify the secretion of signaling molecules from single cells, and investigate cellular communication. In 2015, Sarkar et al. demonstrated an array-based droplet device that allowed monitoring of nanolitre-sized droplets for T-cell activation longitudinally right from the onset of activation (161). Their results suggested that the activation of single T-cells is faster when cells come in contact with dendritic cells in comparison to other activation methods. Furthermore, they developed a method to probe into the potentially heterogeneous cytolytic behavior of human NK cells (162). They demonstrated a 100% killing efficiency of NK cells, which is in contrast to earlier findings by various groups performed either in bulk or single cell (105, 163).

**Figure 5:**
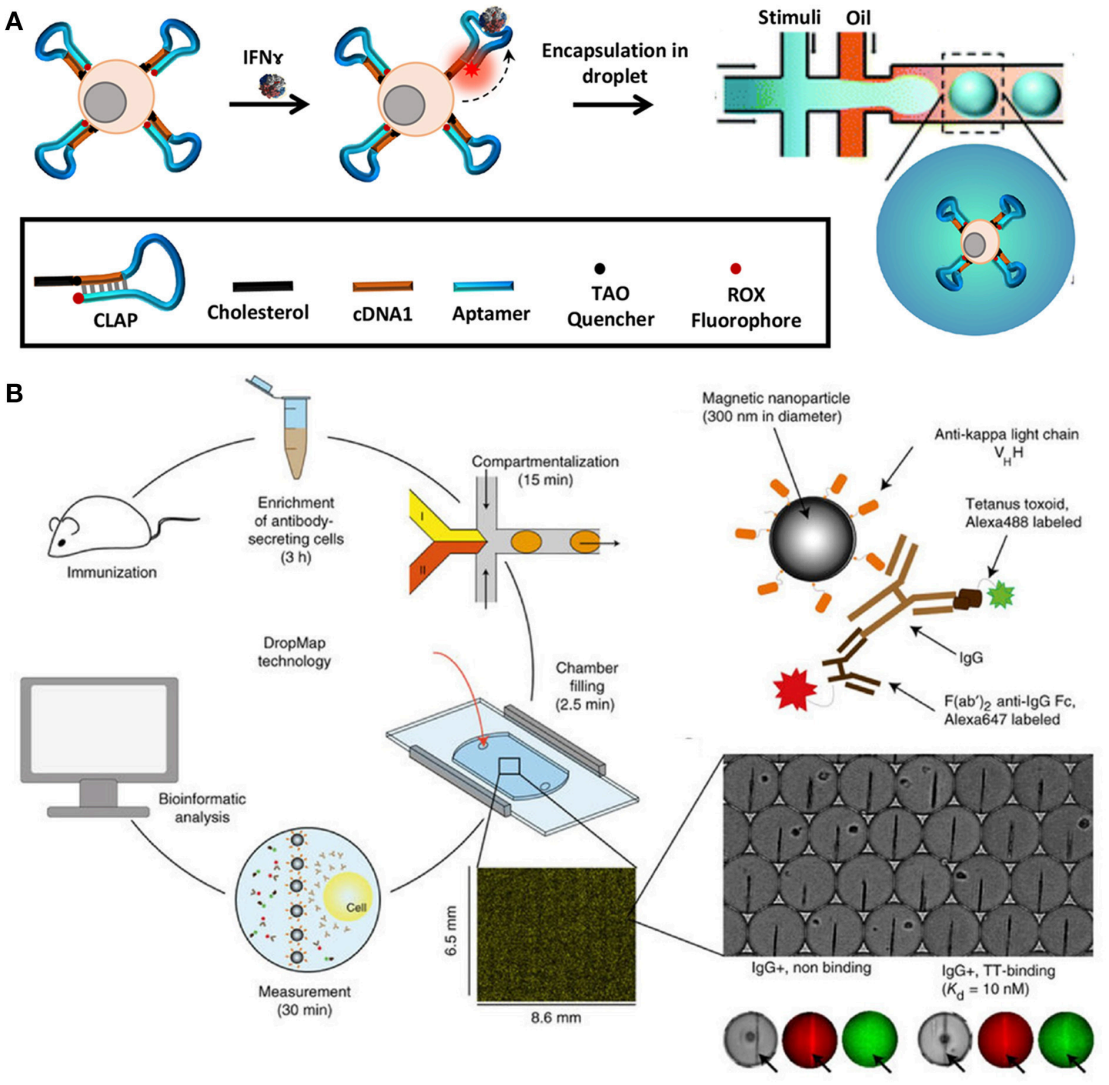
Droplet microfluidics is a very versatile tool that allows single-cell analysis of immune cells in a noise-free environment. The cells are often paired with functionalized beads or sensors such as aptamer sensors for quantification of secreted molecules and proteins. Droplet microfluidics is combined with flow cytometry, mass cytometry, and automated microscopy for downstream analysis. **(A)** Adapted from Qiu et al. (165). **(B)** Adapted from Eyer et al. (166). Reproduced with permission from Springer Nature.

In order to quantify secreted molecules, cells are paired with functionalised beads or other sensing molecules to capture target analytes during incubation, prior to analysis (164). The droplet interface ensures that encapsulated cells are shielded from external factors that might influence their secretory behavior. Concurrently, this interface in combination with the small droplet volume, confines secreted molecules within the droplet resulting in increased sensitivity. Qiu et al. employed aptamer-based DNA sensors to quantify IFNγ secretion by encapsulating single T-cells in droplets followed by flow-cytometric and microscopic analysis [Figure 5A; (165)]. This study demonstrated the versatility of droplet microfluidics to be integrated with multiple detection methodologies. In another recent study, Eyer et al. used DropMap technology for phenotyping IgG secreting plasma cells at single-cell level [Figure 5B; (166)]. In this study, they paired antibody secreting cells with multiple paramagnetic functionalised nanoparticles that capture target antibodies in picolitre sized droplets. For the purpose of analysis, the generated droplets were immobilized in a glass observation chamber to measure fluorescence intensity of each droplet and to quantify secreted antibodies to map different plasma cell phenotypes. With this technology it is possible to monitor and quantify antibody secretion by encapsulated cells in droplets real time.

**Figure 6:**
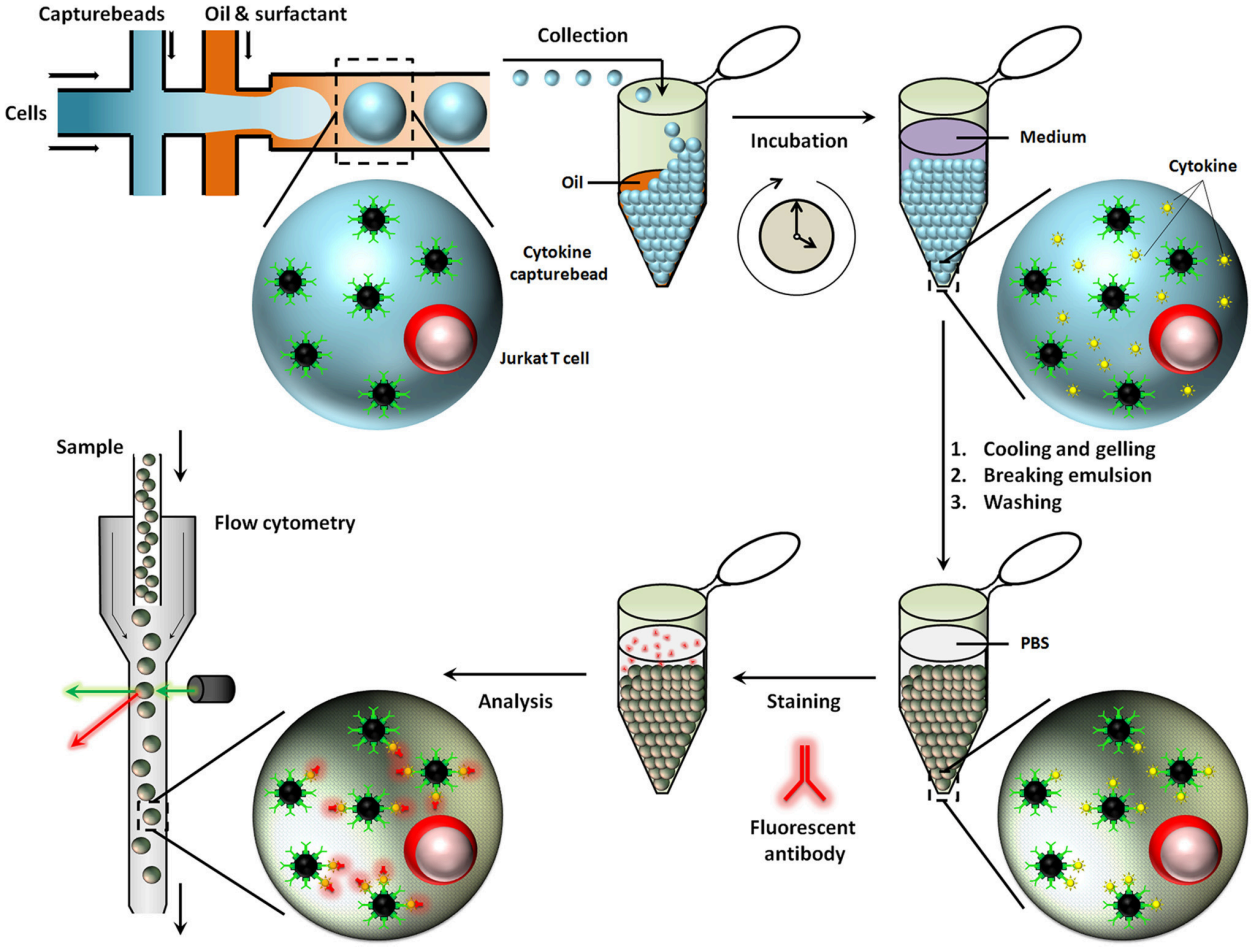
Hydrogel agarose gel droplets used in the Huck laboratory for measurement of cytokine secretion. The advantage of hydrogel droplets is that it allows washing steps for immunoassays. Also, cells encapsulated in hydrogel droplets can directly be analyzed by cytometry. Figure adapted from Chokkalingam et al. (167).

Besides aqueous based droplets, hydrogel agarose can also be used to create droplets in oil phase, which allows washing steps and permits staining with antibodies within droplets by slow diffusion. This conceptual advantage of using hydrogel based droplets was exploited in the Huck laboratory, where agarose droplets were used for encapsulation of Jurkat T-cells to capture multiple cytokines on functionalised beads and used to demonstrate cellular heterogeneity and mapping cellular subsets [Figure 6; (167)]. Generally, for cytometry, droplets need to be broken to retrieve cells and beads. On the contrary, cells and beads encapsulated in hydrogels can be analyzed directly with flow cytometry, preventing loss of cells and saving significant amounts of time.

Recently, researchers have also implemented protocols for single-cell sequencing in droplet microfluidics (168). In 2015, Macosko et al. developed the Drop-seq technology where the transcriptomics of thousands of retinal cells were analyzed in droplets using barcoded microparticles (169). Later, the Abate lab also demonstrated the genomic sequencing of more than fifty thousand cells at single-cell level in agarose microgels (170). Single-cell sequencing allows researchers to identify the differences in cellular behavior and understand the functionalities of individual cells, which assists in decoding immune cell heterogeneity (171). Genomic amplication for sequencing can be performed in droplet microfluidics with high accuracy and specificity in a massively parallel fashion (168). The work of Shahi et al. demonstrated the efficiency of droplet microfluidics to profile protein secretion by single immune cells using a high-throughput droplet-microfluidic barcoding technique, Abseq (172). This microfluidic device was integrated with functions to amplify DNA in nanolitre sized droplets to allow more than tens of thousands of cells to be analyzed in parallel.

Together, all these studies highlight the role of droplet microfluidics in single-cell analysis of immune cells. Droplet-based microfluidics is a highly versatile and flexible technology and is widely applicable in multiple realms of immunology. The ability to carry out high-throughput analysis of hundreds to thousands of individual immune cells and paired immune cells in a parallel manner makes droplet microfluidics a highly reliable and popular single-cell analysis tool.

### Strengths and weaknesses of microfluidic technologies

The motivation for miniaturization was driven by the requirement to acquire more information from single cells at higher resolution. Flow cytometric analysis allows for sampling cell populations in time but fails to provide dynamic information from single immune cells. Also, high costs of the equipment and infrastructure for mass cytometers often limits the usage of this technology. To compensate for the drawbacks of the cytometric analysis and gather more temporal information on the behavior of single immune cells, microsystems and microfluidics gained popularity. Innovation in microsystems and microfluidics facilitated the integration of numerous complex functions on-chip that were earlier not feasible or demanded a lot of manual labor. At the same time, the ability of these platforms to dynamically acquire information from immune cells and monitor immune cell activities real time made them popular among researchers.

Nanowells and microfluidic chips with hydrodynamic cell traps are the simplest examples of miniaturization that, because of ease of fabrication and operation, are frequently used for decoding immune cell behavior and intercellular communication. These high-throughput analysis platforms allow both real-time and end-point measurements and can facilitate one to one cell pairing for decoding communication between immune cells, e.g., for monitoring cytotoxic cellular function. However, microfluidic chips with cell traps are more efficient in achieving desired pairing efficiencies in comparison to nanowells. These platforms are limited by their ability to replicate the dynamically variable immune cell microenvironment in which immune cells work. Also, for more efficient single-cell level analysis of immune cells it is essential that cells are isolated and analyzed in a noise-free environment to negate the effects of paracrine communication from neighboring cells.

Valve- and droplet-based microfluidics have realized the aforementioned key requirements and have been able to circumvent the drawbacks of other single-cell tools. Both these platforms have the ability to compartmentalize cells in a closed environment to understand cellular behavior with high sensitivity. One of the key advantages of programmable valve-based microfluidics is that it allows the replication of dynamic immune cell microenvironments with high precision for delivering input stimuli in forms of pulsatile bursts. Although the process of fabrication and experimental setup for such devices is fairly complex and time-consuming, automation and reproducibility compensates for the drawbacks of these designs (132). Also, the throughput of mLSI designs is often low to medium, but has the capacity to be increased by scaling.

For high-throughput analysis of immune cells, droplet-based microfluidics is preferred. Easy to design and implement for multiple applications, it allows the isolation of single immune cells in droplets for analysis in an isolated system. Small compartment size and very low droplet volumes makes this system highly sensitive by preventing the loss of stimuli and secreted molecules from the system. Further, this system also facilitates encapsulation of multiple cells in a closed environment to understand immune cell communication, e.g., for cytotoxic behavior. While droplet microfluidics is a well-established tool in the single-cell analysis community, it often finds limited applications with use of primary, rare immune cells because of the difficulties faced during seeding of cells. Traditional cell seeding methods often lead to a loss of the cells because of attachment or sedimentation, and when cells are already rare in population, it is difficult to obtain high encapsulation rates (173). Encapsulation of cells in droplets is random and relies heavily on Poisson statistics (174). To overcome the limitations of Poisson statistics alternative cell seeding methods as well as use of external physical forces are required to ensure desired cell distribution in droplets (175, 176). The designs discussed in this review, each with their own set of advantages and disadvantages, have been widely implemented for several single cell studies to enhance our understanding of various immune cell functions (Table 1).

**Table 1:** Table summarizing different single-cell analysis tools discussed in this review in terms of their advantages, disadvantages, and applications.

	**Research applications**	**Advantages**	**Limitation**	**Commercial vendors**	**References**
Cytometry	Cytokine and surface markers Signaling and activation dynamics Cytotoxicity Immunophenotyping Sorting	High throughput Quick to run (on the order of several hours) Ability to sort cells based on expression of molecules	Spectral overlaps Expensive Not possible to replicate dynamic cellular microenvironment Static snapshot over time	BD Biosciences, MilliporeSigma, Miltenyi Biotec, Thermo Fisher Scientific	(177), (178), (179)
**MICROSYSTEMS**					
Nano wells	Antibody and drug screening Activation dynamics Cytotoxicity Inter and intra cellular communication Cytokines and signaling molecules release	High-throughput Possibility to recover cells from the wells Simple to use Multiplexing and real time monitoring Quantitative analysis of proteins Possibility to pair multiple cells in each well	Seeding cells under the effect of gravity lowers the efficiency of cell loading Manual operation Limited control over the fluidic and cellular microenvironment Random distribution of cells in wells	μFluidix, microfluidic ChipShop	(76), (77), (79), (80), (163), (55), (78)
**MICROFLUIDICS**					
Trap-Based	Inter and intracellular communication Cytotoxicity Signaling and activation dynamics Cytokines and signaling molecules release	Efficient cell pairing and fusion Ability to monitor cellular behavior, from the point of cellular interaction and contact Multiplexing and real time monitoring High throughput	Stimulation of cells in bulk and not on-chip (Juxtacrine and paracrine interactions before loading the cells cannot be ruled out) Controlled on-chip stimulation is not possible Cells are not isolated from each other (The trapped cells and secreted molecules are not completely confined and this can influence the behavior of the neighboring cells by paracrine communication)	μFluidix, microfluidic ChipShop	(104), (105), (106), (96), (109)
Valve-based	Signaling and activation dynamics Cytokines and signaling molecules release Monitor transcription factors activity Migration	Complete automation Replication of dynamic cellular microenvironment Sensitive (addressable cell chambers with very small volume) Low sample volume Both real time monitoring and end-point analysis	Laborious and time-consuming fabrication Not portable (additional equipment for operation) Low to medium throughput	Fluidigm C1	(128), (129), (135), (16), (180), (181), (182)
Droplet-based	Inter and intracellular communication Cytokines and signaling molecules release Activation dynamics Antibody screening Cytotoxicity	High throughput Noise-free cellular microenvironment due to compartmentalization Both real time monitoring and end point analysis Multiple as well as single cell pairing Sensitive (low reaction volume) Small sample and reagent volume	Not possible to replicate dynamic cellular microenvironment Random encapsulation (follows Poisson Distribution) Difficult to incorporate washing steps	μFluidix, microfluidic ChipShop, Dolomite, Fluigent, 10X Genomics (Chromium Controller) BIORAD (ddSEQ)	(144), (162), (164), (166), (167), (161)

*The applications described here are what is presented in this review as well as all the other potential applications of the design for immune cell analysis at single-cell level*.

## Conclusion and future outlook

Single-cell analysis tools have played a major role in enhancing our understanding of the human immune system. Several research groups have focused on technology development to constantly provide novel design alternatives for biological studies. Thereby, it enabled to address complex immunological questions that were earlier not possible with conventional bulk methods and resulted in identification of heterogeneous immune cell behavior, discovery of new immune cell subsets, and understanding how single immune cells drive population responses. Single-cell analysis facilitated the design and development of new diagnostic tools, personalized medicines, and immunotherapies for treatment of cancer, immunosuppressive diseases, and autoimmune disorders (183–186). For example, single-cell studies recognized specific signaling pathways within individual immune cells that were suppressed in a tumor microenvironment (187). Identification of such immunosuppressive signaling pathways, molecules, and individual immune cells improves the design of treatment modalities aimed at targeting cells and activation of the suppressed signaling pathways to fight cancer, infectious- and auto-immune diseases (184, 188). For development of vaccines, it is critical to understand how specific antigens induce effective immunization. Novel vaccines with higher clinical efficacy can be developed using results from antibody screening and quantification experiments at single-cell level (149, 166). Also, quantification of signaling molecules at single-cell level provides information on new pathways for development of sensitive diagnostic tools that can provide faster and accurate results in comparison to traditional laboratory methods (189).

Developments in the field of microsystems and microfluidics have been ongoing for more than a decade and continues to grow. As our understanding of the human immune systems deepens, more questions arise to decode the complexity of our system. To cater to these questions, technology continues to evolve. The designs discussed in this review were limited to applications in single-cell analysis of immune cells. However, there are several established and on-going design developments in single-cell research for multiple biological applications that can be easily modified and implemented for better understanding of the immune system. As an example, the microfluidic droplet system published by Shembekar et al. can be modified for use with primary B-cells for antibody screening (149). Furthermore, a droplet microfluidic system integrated with Protein Assay via Induced Gene Expression (PAIGE) can be used for quantification of secretory molecules at single-cell level (190). Microfluidic chips can be also be used to study immunosurveillance and migration of immune cells, *in vitro* (191, 192). Finally, there are several other designs that can be integrated with different systems or modified for immune system related research (193–195).

There are multiple small and medium scale companies that have commercialized microfluidics, as individual components or complete analytical system, to promote the integration of microfluidics in multiple laboratories for several single-cell analysis applications. Companies like Dolomite and Fluigent fabricate droplet microfluidic chips that can be bought and directly used in laboratories for research applications. These chips, however, still have to be integrated with downstream analytical methods. Other companies such as μFluidix and microfluidic ChipShop do provide professional facilities to fabricate different types of microfluidic devices as per the user requirements. Sphere Fluidics also provides completely integrated analytical solutions for single-cell research. Further, there are commercial systems integrated with microfluidics that provide complete analytical solutions to research problems. One such example is the C1, developed by FLUIDIGM, that is integrated with microfluidic circuits for transcriptomics at single-cell level to identify heterogeneity among immune cell population. This system provides a fully automated solution to implement experimental protocols with high precision and accuracy. The Chromium Controller by 10X Genomics allows profiling of immune cells and their repertoire at single-cell level with high automation and parallelisation. ddSEQ by BIORAD uses droplet microfluidics for isolation of single cells to provide sequencing solutions at single cell level. This commercial device has multiple applications including assessment of cellular heterogeneity, identification of cellular sub-populations, and functional analysis of immune cells. The aforementioned examples are just a few of the many companies that have commercialized microfluidic technology for research puposes.

Taken together, robust technology for decoding cell-cell or cell-pathogen interactions longitudinally and in great detail will revolutionize cell biology and the fields of immunology and cellular immunotherapy in particular. The impact of rapid expansion of single-cell analysis is evident in its great potential for numerous applications, including, but not limited to: cancer research, regenerative medicine, diagnostics, and synthetic biology. We believe that even though technology development might sometimes be an extended process, the ease and cost-effectiveness of microfluidics will boost the integration of this exciting technology in the portfolio of other single-cell assays used in cell biology and immunology related disciplines.

## Author contributions

NSi, NSu, and JT conceived the manuscript and the writing of the manuscript and approved its final content.

### Conflict of interest statement

The authors declare that the research was conducted in the absence of any commercial or financial relationships that could be construed as a potential conflict of interest.

## Abbreviations

IFN-γ: interferon gamma
IL: interleukin
mLSI: microfluidic large scale integration
NF-κB: nuclear factor-κB
NK cells: natural killer cells
PAIGE: protein assay via induced gene expression
PDMS: polydimethylsiloxane
TCR: T-cell receptor
TNF: tumor necrosis factor.

## References

[1] Janeway C A. How the immune system works to protect the host from infection: a personal view. Proc Natl Acad Sci USA (2001) 98:7461–8. 10.1073/pnas.131202998 11390983 PMC34691

[2] Aderem A Ulevitch RJ. Toll-like receptors in the induction of the innate immune response. Nature (2000) 406:782–7. 10.1038/35021228 10963608

[3] Subramanian N Torabi-Parizi P Gottschalk RA Germain RN Dutta B. Network representations of immune system complexity. Wiley Interdiscip Rev Syst Biol Med. (2015) 7:13–38. 10.1002/wsbm.1288 25625853 PMC4339634

[4] Mueller SN Zaid A Carbone FR. Tissue-resident T cells: dynamic players in skin immunity. Front Immunol. (2014) 5:332. 10.3389/fimmu.2014.00332 25076947 PMC4099935

[5] Castellino F Huang AY Altan-Bonnet G Stoll S Scheinecker C Germain RN. Chemokines enhance immunity by guiding naive CD8+T cells to sites of CD4+T cell-dendritic cell interaction. Nature (2006) 440:890–5. 10.1038/nature04651 16612374

[6] Cheroutre H Huang Y. Crosstalk between adaptive and innate immune cells leads to high quality immune protection at the mucosal borders. Adv Exp Med Biol. (2013) 785:43–7. 10.1007/978-1-4614-6217-0_5 23456836 PMC3913074

[7] Rivera A Siracusa MC Yap GS Gause WC. Innate cell communication kick-starts pathogen-specific immunity. Nat Immunol. (2016) 17:356–63. 10.1038/ni.3375 27002843 PMC4949486

[8] Xie J Tato CM Davis MM. How the immune system talks to itself: the varied role of synapses. Immunol Rev. (2013) 251:65–79. 10.1111/imr.12017 23278741 PMC3645447

[9] Satija R Shalek AK. Heterogeneity in immune responses: from populations to single cells. Trends Immunol. (2014) 35:219–29. 10.1016/j.it.2014.03.004 24746883 PMC4035247

[10] Kaech SM Wherry EJ. Heterogeneity and cell-fate decisions in effector and memory CD8+ T cell differentiation during viral infection. Immunity (2007) 27:393–405. 10.1016/j.immuni.2007.08.007 17892848 PMC3431921

[11] O'Garra A. Cytokines induce the development of functionally heterogeneous T helper cell subsets. Immunity (1998) 8:275–83. 10.1016/S1074-7613(00)80533-6 9529145

[12] Papalexi E Satija R. Single-cell RNA sequencing to explore immune cell heterogeneity. Nat Rev Immunol. (2018) 18:35–45. 10.1038/nri.2017.76 28787399

[13] Chattopadhyay PK Gierahn TM Roederer M Love JC. Single-cell technologies for monitoring immune systems. Nat Immunol. (2014) 15:128–35. 10.1038/ni.2796 24448570 PMC4040085

[14] Novo P Dell'Aica M Janasek D Zahedi RP. High spatial and temporal resolution cell manipulation techniques in microchannels. Analyst (2016) 141:1888–1905. 10.1039/C6AN00027D 26891209

[15] Kellogg RA Tian C Lipniacki T Quake SR Tay S. Digital signaling decouples activation probability and population heterogeneity. Elife (2015) 4:e08931. 10.7554/eLife.08931 26488364 PMC4608393

[16] Ma C Fan R Ahmad H Shi Q Comin-Anduix B Chodon T. A clinical microchip for evaluation of single immune cells reveals high functional heterogeneity in phenotypically similar T cells. Nat Med. (2011) 17:738–43. 10.1038/nm.2375 21602800 PMC3681612

[17] Kirschbaum M Jaeger MS Duschl C Correlating short-term Ca2+ responses with long-term protein expression after activation of single T cells. Lab Chip (2009) 9:3517–25. 10.1039/b911865a 20024031

[18] Hemmer B Nessler S Zhou D Kieseier B Hartung HP Immunopathogenesis and immunotherapy of multiple sclerosis. Nat Clin Pract Neurol. (2006) 2:201–11. 10.1038/ncpneuro0154 16932551

[19] Ma X Hui H Jin Y Dong D Liang X Yang X. Enhanced immunotherapy of SM5-1 in hepatocellular carcinoma by conjugating with gold nanoparticles and its *in vivo* bioluminescence tomographic evaluation. Biomaterials (2016) 87:46–56. 10.1016/j.biomaterials.2016.02.007 26897539

[20] Li Y Fang M Zhang J Wang J Song Y Shi J. Hydrogel dual delivered celecoxib and anti-PD-1 synergistically improve antitumor immunity. Oncoimmunology (2016) 5:e1074374. 10.1080/2162402X.2015.1074374 27057439 PMC4801446

[21] Esch MB King TL Shuler ML. The role of body-on-a-chip devices in drug and toxicity studies. Annu Rev Biomed Eng. (2011) 13:55–72. 10.1146/annurev-bioeng-071910-124629 21513459

[22] Hassell BA Goyal G Lee E Sontheimer-Phelps A Levy O Chen CS. Human organ chip models recapitulate orthotopic lung cancer growth, therapeutic responses, and tumor dormancy *in vitro*. Cell Rep. (2017) 21:508–16. 10.1016/j.celrep.2017.09.043 29020635

[23] Lanz HL Saleh A Kramer B Cairns J Ng CP Yu J. Therapy response testing of breast cancer in a 3D high-throughput perfused microfluidic platform. BMC Cancer (2017) 17:709. 10.1186/s12885-017-3709-3 29096610 PMC5668957

[24] Nagrath S Sequist LV Maheswaran S Bell DW Irimia D Ulkus L. Isolation of rare circulating tumour cells in cancer patients by microchip technology. Nature (2007) 450:1235–9. 10.1038/nature06385 18097410 PMC3090667

[25] Chen H Cao B Sun B Cao Y Yang K Lin YS. Highly-sensitive capture of circulating tumor cells using micro-ellipse filters. Sci Rep. (2017) 7:610. 10.1038/s41598-017-00232-6 28377598 PMC5428045

[26] Khoo BL Grenci G Lim YB Lee SC Han J Lim CT. Expansion of patient-derived circulating tumor cells from liquid biopsies using a CTC microfluidic culture device. Nat Protoc. (2018) 13:34–58. 10.1038/nprot.2017.125 29215634

[27] Del Ben F Turetta M Celetti G Piruska A Bulfoni M Cesselli D. A method for detecting circulating tumor cells based on the measurement of single-cell metabolism in droplet-based microfluidics. Angew Chemie (2016) 55:8581–4. 10.1002/anie.201602328 27247024

[28] Zheng G Patolsky F Cui Y Wang WU Lieber CM. Multiplexed electrical detection of cancer markers with nanowire sensor arrays. Nat Biotechnol. (2005) 23:1294–301. 10.1038/nbt1138 16170313

[29] Chikkaveeraiah BV, Mani V, Patel V, Gutkind JS, Rusling JF. Microfluidic electrochemical immunoarray for ultrasensitive detection of two cancer biomarker proteins in serum. Biosens Bioelectron. (2011) 26:4477–83. 10.1016/j.bios.2011.05.005 21632234 PMC3120903

[30] Walford RL Meredith Pa J Cheney KE Immunoengineering: prospects for correction of age-related immunodeficiency states. In: Makinodan T Yunis E, editors. Immunology and Aging. Comprehensive Immunology. Vol. 1 Boston, MA: Springer (1977). p. 183–4.

[31] Singh A Roy K. Immuno-engineering: the next frontier in therapeutics delivery. Adv Drug Deliv Rev. (2017) 114:1–2. 10.1016/j.addr.2017.08.005 28865770

[32] Goldberg MS. Immunoengineering: how nanotechnology can enhance cancer immunotherapy. Cell (2015) 161:201–4. 10.1016/j.cell.2015.03.037 25860604

[33] Xie YQ Wei L Tang L. Immunoengineering with biomaterials for enhanced cancer immunotherapy. Wiley Interdiscip Rev Nanomedicine Nanobiotechnol. (2018) 10:e1506. 10.1002/wnan.1506 29333729

[34] Weiden J Tel J Figdor CG. Synthetic immune niches for cancer immunotherapy. Nat Rev Immunol. (2018) 18:212–9. 10.1038/nri.2017.89 28853444

[35] Davis MM Tato CM Furman D. Systems immunology: just getting started. Nat Immunol. (2017) 18:725–32. 10.1038/ni.3768 28632713 PMC5790187

[36] Rosenberg SA Yang JC Restifo NP. Cancer immunotherapy: moving beyond current vaccines. Nat Med. (2004) 10:909–15. 10.1038/nm1100 15340416 PMC1435696

[37] Papaioannou NE Beniata OV Vitsos P Tsitsilonis O Samara P. Harnessing the immune system to improve cancer therapy. Ann Transl Med. (2016) 4:261. 10.21037/atm.2016.04.01 27563648 PMC4971375

[38] Jackson HJ Rafiq S Brentjens RJ. Driving CAR T-cells forward. Nat Rev Clin Oncol. (2016) 13:370–83. 10.1038/nrclinonc.2016.36 27000958 PMC5529102

[39] Turtle CJ Riddell SR. Artificial antigen-presenting cells for use in adoptive immunotherapy. Cancer J. (2011) 16:374–81. 10.1097/PPO.0b013e3181eb33a6 20693850 PMC2929753

[40] Eggermont LJ Paulis LE Tel J Figdor CG. Towards efficient cancer immunotherapy: advances in developing artificial antigen-presenting cells. Trends Biotechnol. (2014) 32:456–65. 10.1016/j.tibtech.2014.06.007 24998519 PMC4154451

[41] Khailaie S Bahrami F Janahmadi M Milanez-Almeida P Huehn J Meyer-Hermann M. A mathematical model of immune activation with a unified self-nonself concept. Front Immunol. (2013) 4:474. 10.3389/fimmu.2013.00474 24409179 PMC3872974

[42] Haessler U Pisano M Wu M Swartz MA. Dendritic cell chemotaxis in 3D under defined chemokine gradients reveals differential response to ligands CCL21 and CCL19. Proc Natl Acad Sci USA (2011) 108:5614–9. 10.1073/pnas.1014920108 21422278 PMC3078419

[43] Junkin M Tay S. Microfluidic single-cell analysis for systems immunology. Lab Chip (2014) 14:1246–60. 10.1039/c3lc51182k 24503696

[44] Jeanbart L Swartz MA. Engineering opportunities in cancer immunotherapy. Proc Natl Acad Sci USA (2015) 112:14467–72. 10.1073/pnas.1508516112 26598681 PMC4664348

[45] Swartz MA Hirosue S Hubbell JA. Engineering approaches to immunotherapy. Sci Transl Med. (2012) 4:148rv9. 10.1126/scitranslmed.3003763 22914624

[46] Fesnak AD June CH Levine BL. Engineered T cells: the promise and challenges of cancer immunotherapy. Nat Rev Cancer (2016) 16:566–81. 10.1038/nrc.2016.97 27550819 PMC5543811

[47] Butler MO Hirano N. Human cell-based artificial antigen-presenting cells for cancer immunotherapy. Immunol Rev. (2014) 257:191–209. 10.1111/imr.12129 24329798 PMC3869003

[48] Delcassian D Sattler S Dunlop IE. T cell immunoengineering with advanced biomaterials. Integr Biol. (2017) 9:211–22. 10.1039/c6ib00233a 28252135 PMC6034443

[49] Toriello NM Douglas ES Thaitrong N Hsiao SC Francis MB Bertozzi CR. Integrated microfluidic bioprocessor for single-cell gene expression analysis. Proc Natl Acad Sci USA (2008) 105:20173–8. 10.1073/pnas.0806355106 19075237 PMC2629289

[50] Daojing Wang SB Single cell analysis: the new frontier in ‘Omics.’ Trends Biotechnol. (2010) 28:281–90. 10.1016/j.tibtech.2010.03.002.Single 20434785 PMC2876223

[51] Newell EW Sigal N Nair N Kidd BA Greenberg HB Davis MM. Combinatorial tetramer staining and mass cytometry analysis facilitate T-cell epitope mapping and characterization. Nat Biotechnol. (2013) 31:623–9. 10.1038/nbt.2593 23748502 PMC3796952

[52] Polikowsky HG Wogsland CE Diggins KE Huse K Irish JM. Cutting edge: redox signaling hypersensitivity distinguishes human germinal center B cells. J Immunol. (2015) 195:1364–7. 10.4049/jimmunol.1500904 26157177 PMC4530023

[53] Liu T Yamaguchi Y Shirasaki Y Shikada K Yamagishi M Hoshino K. Single-cell imaging of caspase-1 dynamics reveals an all-or-none inflammasome signaling response. Cell Rep. (2014) 8:974–82. 10.1016/j.celrep.2014.07.012 25127135

[54] Shalek AK Satija R Shuga J Trombetta JJ Gennert D Lu D. Single-cell RNA-seq reveals dynamic paracrine control of cellular variation. Nature (2014) 510:363–9. 10.1038/nature13437 24919153 PMC4193940

[55] Zaretsky I Polonsky M Shifrut E Reich-Zeliger S Antebi Y Aidelberg G. Monitoring the dynamics of primary T cell activation and differentiation using long term live cell imaging in microwell arrays. Lab Chip (2012) 12:5007–15. 10.1039/c2lc40808b 23072772

[56] Wang J Tham D Wei W Shin YS Ma C Ahmad H Shi Q. Quantitating cell-cell interaction functions with applications to glioblastoma multiforme cancer cells. Nano Lett. (2012) 12:6101–6. 10.1021/nl302748q 23130660 PMC3680341

[57] De Rosa SC Herzenberg LA Herzenberg LA Roederer M. 11-color, 13-parameter flow cytometry: identification of human naive T cells by phenotype, function, and T-cell receptor diversity. Nat Med. (2001) 7:245–8. 10.1038/84701 11175858

[58] Roncador G Brown PJ Maestre L Hue S Martínez-Torrecuadrada JL Ling K-L. Analysis of FOXP3 protein expression in human CD4+CD25+ regulatory T cells at the single-cell level. Eur J Immunol. (2005) 35:1681–91. 10.1002/eji.200526189 15902688

[59] Josefowicz SZ Rudensky A. Control of regulatory T cell lineage commitment and maintenance. Immunity (2009) 30:616–25. 10.1016/j.immuni.2009.04.009 19464984 PMC4410181

[60] Schmitt E Klein M Bopp T. Th9 cells, new players in adaptive immunity. Trends Immunol. (2014) 35:61–8. 10.1016/j.it.2013.10.004 24215739

[61] Eyerich S Eyerich K Pennino D Carbone T Nasorri F Pallotta S. Th22 cells represent a distinct human T cell subset involved in epidermal immunity and remodeling. J Clin Invest. (2009) 119:3573–85. 10.1172/JCI40202 19920355 PMC2786807

[62] Gaublomme JT Yosef N Lee Y Gertner RS Yang LV Wu C. Single-cell genomics unveils critical regulators of Th17 cell pathogenicity. Cell (2015) 163:1400–12. 10.1016/j.cell.2015.11.009 26607794 PMC4671824

[63] Yalçin A Yamanaka YJ Love JC. Analytical technologies for integrated single-cell analysis of human immune responses. Methods Mol Biol. (2012) 853:211–35. 10.1007/978-1-61779-567-1_16 22323150

[64] Kimmerling RJ Lee Szeto G Li JW Genshaft AS Kazer SW Payer KR. A microfluidic platform enabling single-cell RNA-seq of multigenerational lineages. Nat Commun. (2016) 7:10220. 10.1038/ncomms10220 26732280 PMC4729820

[65] Jaitin DA Kenigsberg E Keren-Shaul H Elefant N Paul F Zaretsky I. Massively parallel single-cell RNA-seq for marker-free decomposition of tissues into cell types. Science (2014) 343:776–9. 10.1126/science.1247651 24531970 PMC4412462

[66] Ahn RS Taravati K Lai K Lee KM Nititham J Gupta R. Transcriptional landscape of epithelial and immune cell populations revealed through FACS-seq of healthy human skin. Sci Rep. (2017) 7:1343. 10.1038/s41598-017-01468-y 28465541 PMC5430950

[67] Yan X Wu C Chen T Santos MM Liu CL Yang C. Cathepsin S inhibition changes regulatory T-cell activity in regulating bladder cancer and immune cell proliferation and apoptosis. Mol Immunol. (2017) 82:66–74. 10.1016/j.molimm.2016.12.018 28033540

[68] Korin B Dubovik T Rolls A. Mass cytometry analysis of immune cells in the brain. Nat Protoc. (2018) 13:377–91. 10.1038/nprot.2017.155 29370157

[69] Goetz C Peng LJ Aggeler B Bonnevier J. Phenotyping CD4+ hTh2 cells by flow cytometry: Simultaneous detection of transcription factors, secreted cytokines, and surface markers. Methods Molecul Biol. (2017) 1554:175–84. 10.1007/978-1-4939-6759-9_10 28185190

[70] Lin D Maecker HT. Mass cytometry assays for antigen-specific t cells using CyTOF. Methods Mol Biol. (2018) 1678:37–47. 10.1007/978-1-4939-7346-0_3 29071674 PMC5798871

[71] Dhoble AS Bekal S Dolatowski W Yanz C Lambert KN Bhalerao KD. A novel high-throughput multi-parameter flow cytometry based method for monitoring and rapid characterization of microbiome dynamics in anaerobic systems. Bioresour Technol. (2016) 220:566–71. 10.1016/j.biortech.2016.08.076 27614579

[72] Bendall SC Nolan GP Roederer M Chattopadhyay PK. A deep profiler's guide to cytometry. Trends Immunol. (2012) 33:323–32. 10.1016/j.it.2012.02.010 22476049 PMC3383392

[73] Giesen C Wang HAO Schapiro D Zivanovic N Jacobs A Hattendorf B. Highly multiplexed imaging of tumor tissues with subcellular resolution by mass cytometry. Nat Methods (2014) 11:417–22. 10.1038/nmeth.2869 24584193

[74] Clausell-Tormos J Lieber D Baret JC El-Harrak A Miller OJ Frenz L. Droplet-based microfluidic platforms for the encapsulation and screening of mammalian cells and multicellular organisms. Chem Biol. (2008) 15:427–37. 10.1016/j.chembiol.2008.04.004 18482695

[75] Dove A. Drug screening - beyond the bottleneck. Nat Biotechnol. (1999) 17:859–63. 10.1038/12845 10471925

[76] Love JC Ronan JL Grotenbreg GM Van Der Veen AG Ploegh HL. A microengraving method for rapid selection of single cells producing antigen-specific antibodies. Nat Biotechnol. (2006) 24:703–7. 10.1038/nbt1210 16699501

[77] Torres AJ Hill AS Love JC. Nanowell-based immunoassays for measuring single-cell secretion: characterization of transport and surface binding. Anal Chem. (2014) 86:11562–9. 10.1021/ac4030297 25347613 PMC4255675

[78] Torres AJ Contento RL Gordo S Wucherpfennig KW Love JC. Functional single-cell analysis of T-cell activation by supported lipid bilayer-tethered ligands on arrays of nanowells. Lab Chip (2013) 13:90–9. 10.1039/c2lc40869d 23070211 PMC3522575

[79] Yamanaka YJ Berger CT Sips M Cheney PC Alter G Love JC. Single-cell analysis of the dynamics and functional outcomes of interactions between human natural killer cells and target cells. Integr Biol. (2012) 4:1175–84. 10.1039/c2ib20167d 22945136

[80] An X Sendra VG Liadi I Ramesh B Romain G Haymaker C. Single-cell profiling of dynamic cytokine secretion and the phenotype of immune cells. PLoS ONE (2017) 12:e0181904. 10.1371/journal.pone.0181904 28837583 PMC5570329

[81] Sackmann EK Fulton AL Beebe DJ. The present and future role of microfluidics in biomedical research. Nature (2014) 507:181–9. 10.1038/nature13118 24622198

[82] Young EWK Beebe DJ. Fundamentals of microfluidic cell culture in controlled microenvironments. J Chem Soc. (2010) 39:1036–48. 10.1039/B813328J/Analyst 20179823 PMC2967183

[83] Zhu Q Qiu L Xu Y Li G Mu Y. Single cell digital polymerase chain reaction on self-priming compartmentalization chip. Biomicrofluidics (2017) 11:014109. 10.1063/1.4975192 28191267 PMC5291791

[84] Rodríguez-Ruiz I Babenko V Martínez-Rodríguez S Gavira JA. Protein separation under a microfluidic regime. Analyst (2018) 143:606–19. 10.1039/c7an01568b 29214270

[85] Kim D Wu X Young AT Haynes CL. Microfluidics-based in vivo mimetic systems for the study of cellular biology. Acc Chem Res. (2014) 47:1165–73. 10.1021/ar4002608 24555566 PMC3993883

[86] Yi C Li CW Ji S Yang M Microfluidics technology for manipulation and analysis of biological cells. Anal Chim Acta (2006) 560:1–23. 10.1016/j.aca.2005.12.037 30442405

[87] Meyvantsson I Beebe DJ. Cell culture models in microfluidic systems. Annu Rev Anal Chem. (2008) 1:423–9. 10.1146/annurev.anchem.1.031207.113042 20636085

[88] Deng Y Zhang Y Sun S Wang Z Wang M Yu B. An integrated microfluidic chip system for single-cell secretion profiling of rare circulating tumor cells. Sci Rep. (2014) 4:7499. 10.1038/srep07499 25511131 PMC4266859

[89] Hultström J Manneberg O Dopf K Hertz HM Brismar H Wiklund M. Proliferation and viability of adherent cells manipulated by standing-wave ultrasound in a microfluidic chip. Ultrasound Med Biol. (2007) 33:145–51. 10.1016/j.ultrasmedbio.2006.07.024 17189057

[90] Diercks AH Ozinsky A Hansen CL Spotts JM Rodriguez DJ Aderem A. A microfluidic device for multiplexed protein detection in nano-liter volumes. Anal Biochem. (2009) 386:30–5. 10.1016/j.ab.2008.12.012 19133224 PMC2678059

[91] Lecault V White AK Singhal A Hansen CL. Microfluidic single cell analysis: from promise to practice. Curr Opin Chem Biol. (2012) 16:381–90. 10.1016/j.cbpa.2012.03.022 22525493

[92] Sia SK Whitesides GM. Microfluidic devices fabricated in poly(dimethylsiloxane) for biological studies. Electrophoresis (2003) 24:3563–76. 10.1002/elps.200305584 14613181

[93] Kane RS Takayama S Ostuni E Ingber DE Whitesides GM. Patterning proteins and cells using soft lithography. Biomaterials (1999) 20:2363–76. 10.1016/S0142-9612(99)00165-9 10614942

[94] Bhagat AAS Jothimuthu P Papautsky I. Photodefinable polydimethylsiloxane (PDMS) for rapid lab-on-a-chip prototyping. Lab Chip (2007) 7:1192–7. 10.1039/b704946c 17713619

[95] Thorsen T Maerkl SJ Quake SR Hadd AG Jacobson SC Ramsey JM. Microfluidic large-scale integration. Science (2002) 298:580–4. 10.1126/science.1076996 12351675

[96] Kobel S Valero A Latt J Renaud P Lutolf M. Optimization of microfluidic single cell trapping for long-term on-chip culture. Lab Chip (2010) 10:857–63. 10.1039/b918055a 20300672

[97] Zhou Y Basu S Wohlfahrt KJ Lee SF Klenerman D Laue ED Seshia AA. A microfluidic platform for trapping, releasing and super-resolution imaging of single cells. Sensors Actuators, B Chem. (2016) 232:680–91. 10.1016/j.snb.2016.03.131 27594767 PMC4872524

[98] Di Carlo D Aghdam N Lee LP. Single-cell enzyme concentrations, kinetics, and inhibition analysis using high-density hydrodynamic cell isolation arrays. Anal Chem. (2006) 78:4925–30. 10.1021/ac060541s 16841912

[99] Faley SL Copland M Wlodkowic D Kolch W Seale KT Wikswo JP. Microfluidic single cell arrays to interrogate signalling dynamics of individual, patient-derived hematopoietic stem cells. Lab Chip (2009) 9:2659–64. 10.1039/b902083g 19704981

[100] Gossett DR Weaver WM Ahmed NS Di Carlo D. Sequential array cytometry: multi-parameter imaging with a single fluorescent channel. Ann Biomed Eng. (2011) 39:1328–34. 10.1007/s10439-010-0199-8 21136165 PMC3069325

[101] Huebner A Bratton D Whyte G Yang M deMello AJ Abell C. Static microdroplet arrays: a microfluidic device for droplet trapping, incubation and release for enzymatic and cell-based assays. Lab Chip (2009) 9:692–8. 10.1039/B813709A 19224019

[102] Li ZG Liu AQ Klaseboer E Zhang JB Ohl CD. Single cell membrane poration by bubble-induced microjets in a microfluidic chip. Lab Chip (2013) 13:1144–50. 10.1039/c3lc41252k 23364762

[103] Skelley AM Kirak O Suh H Jaenisch R Voldman J. Microfluidic control of cell pairing and fusion. Nat Methods (2009) 6:147–52. 10.1038/nmeth.1290 19122668 PMC3251011

[104] Dura B Dougan SK Barisa M Hoehl MM Lo CT Ploegh HL. Profiling lymphocyte interactions at the single-cell level by microfluidic cell pairing. Nat Commun. (2015) 6:5940. 10.1038/ncomms6940 25585172

[105] Dura B Servos MM Barry RM Ploegh HL Dougan SK Voldman J. Longitudinal multiparameter assay of lymphocyte interactions from onset by microfluidic cell pairing and culture. Proc Natl Acad Sci USA (2016) 113:E3599–608. 10.1073/pnas.1515364113 27303033 PMC4932925

[106] Dura B Liu Y Voldman J. Deformability-based microfluidic cell pairing and fusion. Lab Chip (2014) 14:2783–90. 10.1039/c4lc00303a 24898933

[107] Valeyev NV Hundhausen C Umezawa Y Kotov NV Williams G Clop A. A systems model for immune cell interactions unravels the mechanism of inflammation in human skin. PLoS Comput Biol. (2010) 6:e1001024. 10.1371/journal.pcbi.1001024 21152006 PMC2996319

[108] Ng CT Snell LM Brooks DG Oldstone MBA. Networking at the level of host immunity: immune cell interactions during persistent viral infections. Cell Host Microbe (2013) 13:652–64. 10.1016/j.chom.2013.05.014 23768490 PMC3713852

[109] Hoehl MM Dougan SK Ploegh H Voldman J Massively parallel microfluidic cell-pairing platform for the statistical study of immunological cell-cell interactions. In: 15th International Conference on Miniaturized Systems for Chemistry and Life Sciences (Seattle, WA).

[110] Araci IE Brisk P. Recent developments in microfluidic large scale integration. Curr Opin Biotechnol. (2014) 25:60–8. 10.1016/j.copbio.2013.08.014 24484882

[111] Fidalgo LM Maerkl SJ. A software-programmable microfluidic device for automated biology. Lab Chip (2011) 11:1612–9. 10.1039/c0lc00537a 21416077

[112] Taylor RJ Falconnet D Niemisto A Ramsey SA Prinz S Shmulevich I. Dynamic analysis of MAPK signaling using a high-throughput microfluidic single-cell imaging platform. Proc Natl Acad Sci USA (2009) 106:3758–63. 10.1073/pnas.0813416106 19223588 PMC2644260

[113] Fan HC Wang J Potanina A Quake SR. Whole-genome molecular haplotyping of single cells. Nat Biotechnol. (2011) 29:51–9. 10.1038/nbt.1739 21170043 PMC4098715

[114] Gomez-Sjoberg R Leyrat AA Pirone DM Chen CS Quake SR. Versatile, fully automated, microfluidic cell culture system. Anal Chem. (2007) 79:8557–63. 10.1021/ac071311w 17953452

[115] Zhong JF Chen Y Marcus JS Scherer A Quake SR Taylor CR. A microfluidic processor for gene expression profiling of single human embryonic stem cells. Lab Chip (2008) 8:68–74. 10.1039/b712116d 18094763 PMC4110104

[116] Marcus JS Anderson WF Quake SR. Microfluidic single-cell mRNA isolation and analysis. Anal Chem. (2006) 78:3084–9. 10.1021/ac0519460 16642997

[117] Hong JW Studer V Hang G Anderson WF Quake SR. A nanoliter-scale nucleic acid processor with parallel architecture. Nat Biotechnol. (2004) 22:435–9. 10.1038/nbt951 15024389

[118] Hong JW Chen Y Anderson WF Quake SR Molecular biology on a microfluidic chip. J Phys Condens Matter (2006) 18:S691–701. 10.1088/0953-8984/18/18/S14

[119] Ketterer S Hövermann D Guebeli RJ Bartels-Burgahn F Riewe D Altmann T. Transcription factor sensor system for parallel quantification of metabolites on-chip. Anal Chem. (2014) 86:12152–8. 10.1021/ac503269m 25479036

[120] Blazek M Betz C Hall NM Reth M Zengerle R Meier M Proximity ligation assay for high-content profiling of cell signalling pathways on a microfluidic chip. Mol Cell Proteomics (2013) 12:3898–907. 10.1074/mcp.M113.032821 24072685 PMC3861732

[121] Blazek M Santisteban TS Zengerle R Meier M. Analysis of fast protein phosphorylation kinetics in single cells on a microfluidic chip. Lab Chip (2015) 15:726–34. 10.1039/c4lc00797b 25428717

[122] Blackburn MC Petrova E Correia BE Maerkl SJ. Integrating gene synthesis and microfluidic protein analysis for rapid protein engineering. Nucleic Acids Res. (2015) 44:e68. 10.1093/nar/gkv1497 26704969 PMC4838357

[123] Volpetti F Garcia-Cordero J Maerkl SJ. A microfluidic platform for high-throughput multiplexed protein quantitation. PLoS ONE (2015) 10:e0117744. 10.1371/journal.pone.0117744 25680117 PMC4334502

[124] Geertz M Shore D Maerkl SJ. Massively parallel measurements of molecular interaction kinetics on a microfluidic platform. Proc Natl Acad Sci USA (2012) 109:16540–5. 10.1073/pnas.1206011109 23012409 PMC3478601

[125] Pollard JW Opinion: tumour-educated macrophages promote tumour progression and metastasis. Nat Rev Cancer (2004) 4:71–8. 10.1038/nrc1256 14708027

[126] Sumit M Takayama S Linderman JJ. New insights into mammalian signaling pathways using microfluidic pulsatile inputs and mathematical modeling. Integr Biol. (2017) 9:6–21. 10.1039/C6IB00178E 27868126 PMC5259548

[127] Martinez-Corral R Garcia-Ojalvo J Modeling cellular regulation by pulsatile inputs. Curr Opin Syst Biol. (2017) 3:23–9. 10.1016/j.coisb.2017.03.003

[128] Junkin M Kaestli AJ Cheng Z Jordi C Albayrak C Hoffmann A Tay S. High-content quantification of single-cell immune dynamics. Cell Rep. (2016) 15:411–22. 10.1016/j.celrep.2016.03.033 27050527 PMC4835544

[129] Kaestli AJ Junkin M Tay S. Integrated platform for cell culture and dynamic quantification of cell secretion. Lab Chip (2017) 17:4124–33. 10.1039/C7LC00839B 29094740

[130] Bonizzi G Karin M. The two NF-κB activation pathways and their role in innate and adaptive immunity. Trends Immunol. (2004) 25:280–8. 10.1016/j.it.2004.03.008 15145317

[131] Lee REC Qasaimeh MA Xia X Juncker D Gaudet S. NF-κB signalling and cell fate decisions in response to a short pulse of tumour necrosis factor. Sci Rep. (2016) 6:39519. 10.1038/srep39519 28004761 PMC5177917

[132] Kellogg RA Gómez-Sjöberg R Leyrat AA Tay S. High-throughput microfluidic single-cell analysis pipeline for studies of signaling dynamics. Nat Protoc. (2014) 9:1713–26. 10.1038/nprot.2014.120 24967621

[133] Tay S Hughey JJ Lee TK Lipniacki T Quake SR Covert MW Single-cell NF-B dynamics reveal digital activation and analogue information processing. Nature (2010) 466:267–71. 10.1038/nature09145 20581820 PMC3105528

[134] Rockel S Geertz M Maerkl SJ. MITOMI: a microfluidic platform for *in vitro* characterization of transcription factor-DNA interaction. Methods Mol Biol. (2012) 786:97–114. 10.1007/978-1-61779-292-2_6 21938622

[135] Frank T Tay S. Automated co-culture system for spatiotemporal analysis of cell-to-cell communication. Lab Chip (2015) 15:2192–200. 10.1039/C5LC00182J 25892510

[136] Nossal GVJ Lederberg J. Antibody production by single cells. Nature (1958) 181:1419–20. 13552693 10.1038/1811419a0

[137] Jakiela S Kaminski TS Cybulski O Weibel DB Garstecki P. Bacterial growth and adaptation in microdroplet chemostats. Angew Chemie (2013) 52:8908–11. 10.1002/anie.201301524 23832572 PMC3879160

[138] Abalde-Cela S Taladriz-Blanco P De Oliveira MG Abell C. Droplet microfluidics for the highly controlled synthesis of branched gold nanoparticles. Sci Rep. (2018) 8:2440. 10.1038/s41598-018-20754-x 29402918 PMC5799180

[139] Kim SC Clark IC Shahi P Abate AR. Single-Cell RT-PCR in microfluidic droplets with integrated chemical lysis. Anal Chem. (2018) 90:1273–9. 10.1021/acs.analchem.7b04050 29256243 PMC5991602

[140] Chen Q Utech S Chen D Prodanovic R Lin J-M Weitz DA. Controlled assembly of heterotypic cells in a core–shell scaffold: organ in a droplet. Lab Chip (2016) 16:1346–9. 10.1039/C6LC00231E 26999495 PMC4829496

[141] Nuti N Verboket PE Dittrich PS. Multivesicular droplets: a cell model system to study compartmentalised biochemical reactions. Lab Chip (2017) 17:3112–9. 10.1039/C7LC00710H 28813055 PMC5642647

[142] Küster SK Pabst M Zenobi R Dittrich PS. Screening for protein phosphorylation using nanoscale reactions on microdroplet arrays. Angew Chem. (2015) 54:1671–5. 10.1002/anie.201409440 25504774

[143] Klein AM Mazutis L Akartuna I Tallapragada N Veres A Li V Peshkin L. Droplet barcoding for single-cell transcriptomics applied to embryonic stem cells. Cell (2015) 161:1187–201. 10.1016/j.cell.2015.04.044 26000487 PMC4441768

[144] Debs BE Utharala R Balyasnikova IV Griffiths AD Merten CA. Functional single-cell hybridoma screening using droplet-based microfluidics. Proc Natl Acad Sci USA (2012) 109:11570–5. 10.1073/pnas.1204514109 22753519 PMC3406880

[145] Guan Z Zou Y Zhang M Lv J Shen H Yang P. A highly parallel microfluidic droplet method enabling single-molecule counting for digital enzyme detection. Biomicrofluidics (2014) 8:014110. 10.1063/1.4866766 24753730 PMC3977795

[146] Park JW Na SC Nguyen TQ Paik SM Kang M Hong D. Live cell imaging compatible immobilization of Chlamydomonas reinhardtii in microfluidic platform for biodiesel research. Biotechnol Bioeng. (2015) 112:494–501. 10.1002/bit.25453 25220860

[147] Pessi J Santos HA Miroshnyk I Joukoyliruusi Weitz DA Mirza S. Microfluidics-assisted engineering of polymeric microcapsules with high encapsulation efficiency for protein drug delivery. Int J Pharm. (2014) 472:82–7. 10.1016/j.ijpharm.2014.06.012 24928131

[148] Shuga J Zeng Y Novak R Lan Q Tang X Rothman N. Single molecule quantitation and sequencing of rare translocations using microfluidic nested digital PCR. Nucleic Acids Res. (2013) 41:e159. 10.1093/nar/gkt613 23873959 PMC3763562

[149] Shembekar N Hu H Eustace D Merten CA. Single-cell droplet microfluidic screening for antibodies specifically binding to target cells. Cell Rep. (2018) 22:2094–106. 10.1016/j.celrep.2018.01.071 29466744 PMC5842027

[150] Rakszewska A Tel J Chokkalingam V Huck WT One drop at a time: toward droplet microfluidics as a versatile tool for single-cell analysis. NPG Asia Mater (2014) 6:e133 10.1038/am.2014.86

[151] Churski K Kaminski TS Jakiela S Kamysz W Baranska-Rybak W Weibel DB. Rapid screening of antibiotic toxicity in an automated microdroplet system. Lab Chip (2012) 12:1629–37. 10.1039/c2lc21284f 22422170

[152] Garstecki P Fuerstman MJ Stone HA Whitesides GM. Formation of droplets and bubbles in a microfluidic T-junction—scaling and mechanism of break-up. Lab Chip (2006) 6:437–446. 10.1039/b510841a 16511628

[153] Seo M Paquet C Nie Z Xu S Kumacheva E Microfluidic consecutive flow-focusing droplet generators. Soft Matter (2007) 3:986–92. 10.1039/b700687j 32900048

[154] Pit AM Duits MHG Mugele F Droplet manipulations in two phase flow microfluidics. Micromachines (2015) 6:1768–93. 10.3390/mi6111455

[155] Chen X Ren CL A microfluidic chip integrated with droplet generation, pairing, trapping, merging, mixing and releasing. RSC Adv. (2017) 7:16738–50. 10.1039/C7RA02336G

[156] Padmanabhan S Misteli T DeVoe DL. Controlled droplet discretization and manipulation using membrane displacement traps. Lab Chip (2017) 17:3717–24. 10.1039/C7LC00910K 28990023 PMC7900922

[157] Lorenz RM Edgar JS Jeffries GDM Chiu DT. Microfluidic and optical systems for the on-demand generation and manipulation of single femtoliter-volume aqueous droplets. Anal Chem. (2006) 78:6433–9. 10.1021/ac060748l 16970318

[158] Franke T Abate AR Weitz DA Wixforth A. Surface acoustic wave (SAW) directed droplet flow in microfluidics for PDMS devices. Lab Chip (2009) 9:2625–7. 10.1039/b906819h 19704975

[159] Chiang MY Hsu YW Hsieh HY Chen SY Fan SK. Constructing 3D heterogeneous hydrogels from electrically manipulated prepolymer droplets and crosslinked microgels. Sci Adv. (2016) 2:e1600964. 10.1126/sciadv.1600964 27819046 PMC5091359

[160] Collins DJ Neild A deMello A Liu A-Q Ai Y. The Poisson distribution and beyond: methods for microfluidic droplet production and single cell encapsulation. Lab Chip (2015) 15:3439–59. 10.1039/C5LC00614G 26226550

[161] Sarkar S Motwani B Sabhachandani P Cohen N Konry T. T cell dynamic activation and functional analysis in nanoliter droplet microarray. J Clin Cell Immunol. (2015) 6:334. 10.4172/2155-9899.1000334 26613065 PMC4657871

[162] Sarkar S Sabhachandani P Ravi D Potdar S Purvey S Beheshti A. Dynamic analysis of human natural killer cell response at single-cell resolution in B-Cell Non-Hodgkin Lymphoma. Front Immunol. (2017) 8:1736. 10.3389/fimmu.2017.01736 29312292 PMC5735063

[163] Guldevall K Brandt L Forslund E Olofsson K Frisk TW Olofsson PE. Microchip screening platform for single cell assessment of NK cell cytotoxicity. Front Immunol. (2016) 7:119. 10.3389/fimmu.2016.00119 27092139 PMC4820656

[164] Konry T Dominguez-Villar M Baecher-Allan C Hafler DA Yarmush ML. Droplet-based microfluidic platforms for single T cell secretion analysis of IL-10 cytokine. Biosens Bioelectron. (2011) 26:2707–10. 10.1016/j.bios.2010.09.006 20888750 PMC3141325

[165] Qiu L Wimmers F Weiden J Heus HA Tel J Figdor CG. A membrane-anchored aptamer sensor for probing IFNγ secretion by single cells. Chem Commun. (2017) 53:8066–9. 10.1039/c7cc03576d 28675396

[166] Eyer K Doineau RCL Castrillon CE Briseño-Roa L Menrath V Mottet G. Single-cell deep phenotyping of IgG-secreting cells for high-resolution immune monitoring. Nat Biotechnol. (2017) 35:977–82. 10.1038/nbt.3964 28892076

[167] Chokkalingam V Tel J Wimmers F Liu X Semenov S Thiele J. Probing cellular heterogeneity in cytokine-secreting immune cells using droplet-based microfluidics. Lab Chip (2013) 13:4740–4. 10.1039/c3lc50945a 24185478

[168] Hosokawa M Nishikawa Y Kogawa M Takeyama H. Massively parallel whole genome amplification for single-cell sequencing using droplet microfluidics. Sci Rep (2017) 7:5199. 10.1038/s41598-017-05436-4 28701744 PMC5507899

[169] Macosko EZ Basu A Satija R Nemesh J Shekhar K Goldman M. Highly parallel genome-wide expression profiling of individual cells using nanoliter droplets. Cell (2015) 161:1202–14. 10.1016/j.cell.2015.05.002 26000488 PMC4481139

[170] Lan F Demaree B Ahmed N Abate AR. Single-cell genome sequencing at ultra-high-throughput with microfluidic droplet barcoding. Nat Biotechnol. (2017) 35:640–6. 10.1038/nbt.3880 28553940 PMC5531050

[171] Eberwine J Sul JY Bartfai T Kim J. The promise of single-cell sequencing. Nat Methods (2014) 11:25–7. 10.1038/nmeth.2769 24524134

[172] Shahi P Kim SC Haliburton JR Gartner ZJ Abate AR. Abseq: ultrahigh-throughput single cell protein profiling with droplet microfluidic barcoding. Sci Rep. (2017) 7:44447. 10.1038/srep44447 28290550 PMC5349531

[173] Mazutis L Gilbert J Ung WL Weitz DA Griffiths AD Heyman JA. Single-cell analysis and sorting using droplet-based microfluidics. Nat Protoc. (2013) 8:870–91. 10.1038/nprot.2013.046 23558786 PMC4128248

[174] Wu L Chen P Dong Y Feng X Liu BF. Encapsulation of single cells on a microfluidic device integrating droplet generation with fluorescence-activated droplet sorting. Biomed Microdevices (2013) 15:553–60. 10.1007/s10544-013-9754-z 23404263

[175] Kemna EWM Schoeman RM Wolbers F Vermes I Weitz DA van den Berg A. High-yield cell ordering and deterministic cell-in-droplet encapsulation using Dean flow in a curved microchannel. Lab Chip (2012) 12:2881–7. 10.1039/c2lc00013j 22688131

[176] Chung MT Núñez D Cai D Kurabayashi K. Deterministic droplet-based co-encapsulation and pairing of microparticles: Via active sorting and downstream merging. Lab Chip (2017) 17:3664–71. 10.1039/c7lc00745k 28967663

[177] Bendall SC Simonds EF Qiu P Amir EAD Krutzik PO Finck R. Single-cell mass cytometry of differential immune and drug responses across a human hematopoietic continuum. Science (2011) 332:687–96. 10.1126/science.1198704 21551058 PMC3273988

[178] Qiu P Simonds EF Bendall SC Gibbs KD Bruggner RV Linderman MD. Extracting a cellular hierarchy from high-dimensional cytometry data with SPADE. Nat Biotechnol. (2011) 29:886–93. 10.1038/nbt.1991 21964415 PMC3196363

[179] Krutzik PO Nolan GP. Fluorescent cell barcoding in flow cytometry allows high-throughput drug screening and signaling profiling. Nat Methods (2006) 3:361–8. 10.1038/nmeth872 16628206

[180] Bao XR Fraser IDC Wall EA Quake SR Simon MI. Variability in G-protein-coupled signaling studied with microfluidic devices. Biophys J. (2010) 99:2414–22. 10.1016/j.bpj.2010.08.043 20959081 PMC2955501

[181] Frank T Tay S. Flow-switching allows independently programmable, extremely stable, high-throughput diffusion-based gradients. Lab Chip (2013) 13:1273–81. 10.1039/c3lc41076e 23386049

[182] Singhal A Haynes CA Hansen CL. Microfluidic measurement of antibody-antigen binding kinetics from low-abundance samples and single cells. Anal Chem. (2010) 82:8671–9. 10.1021/ac101956e 20857931

[183] Armbrecht L Gabernet G Kurth F Hiss JA Schneider G Dittrich PS. Characterisation of anticancer peptides at the single-cell level. Lab Chip (2017) 17:2933–40. 10.1039/c7lc00505a 28736788 PMC6440648

[184] Krieg C Nowicka M Guglietta S Schindler S Hartmann FJ Weber LM. High-dimensional single-cell analysis predicts response to anti-PD-1 immunotherapy. Nat Med. (2018) 24:144–53. 10.1038/nm.4466 29309059

[185] Wiegand A Spindler J Hong FF Shao W Cyktor JC Cillo AR. Single-cell analysis of HIV-1 transcriptional activity reveals expression of proviruses in expanded clones during ART. Proc Natl Acad Sci USA (2017) 114:E3659–68. 10.1073/pnas.1617961114 28416661 PMC5422779

[186] Golumbeanu M Cristinelli S Rato S Munoz M Cavassini M Beerenwinkel N. Single-cell RNA-seq reveals transcriptional heterogeneity in latent and reactivated HIV-infected cells. Cell Rep. (2018) 23:942–50. 10.1016/j.celrep.2018.03.102 29694901

[187] Khandelwal N Breinig M Speck T Michels T Kreutzer C Sorrentino A. A high-throughput RNAi screen for detection of immune-checkpoint molecules that mediate tumor resistance to cytotoxic T lymphocytes. EMBO Mol Med. (2015) 7:450–63. 10.15252/emmm.201404414 25691366 PMC4403046

[188] Marçais A Walzer T. An immunosuppressive pathway for tumor progression. Nat Med. (2018) 24:260–1. 10.1038/nm.4508 29509752

[189] Hou H Zhao Y Li C Wang M Xu X Jin Y. Single-cell pH imaging and detection for pH profiling and label-free rapid identification of cancer-cells. Sci Rep. (2017) 7:1759. 10.1038/s41598-017-01956-1 28496209 PMC5431805

[190] Abbaspourrad A Zhang H Tao Y Cui N Asahara H Zhou Y. Label-free single-cell protein quantification using a drop-based mix-and-read system. Sci Rep. (2015) 5:12756. 10.1038/srep12756 26234416 PMC4522677

[191] Schwarz J Bierbaum V Merrin J Frank T Hauschild R Bollenbach T. A microfluidic device for measuring cell migration towards substrate-bound and soluble chemokine gradients. Sci Rep. (2016) 6:36440. 10.1038/srep36440 27819270 PMC5098208

[192] Jain NG Wong EA Aranyosi AJ Boneschansker L Markmann JF Briscoe DM. Microfluidic mazes to characterize T-cell exploration patterns following activation *in vitro*. Integr Biol. (2015) 7:1423–31. 10.1039/c5ib00146c 26325525 PMC4630132

[193] Eduati F Utharala R Madhavan D Neumann UP Longerich T Cramer T. A microfluidics platform for combinatorial drug screening on cancer biopsies. Nat Commun. (2018) 9:2434. 10.1038/s41467-018-04919-w 29934552 PMC6015045

[194] Garcia-Cordero JL Nembrini C Stano A Hubbell JA Maerkl SJ. A high-throughput nanoimmunoassay chip applied to large-scale vaccine adjuvant screening. Integr Biol. (2013) 5:650–8. 10.1039/c3ib20263a 23443913

[195] Woodruff K Maerkl SJ. Microfluidic module for real-time generation of complex multimolecule temporal concentration profiles. Anal Chem. (2018) 90:696–701. 10.1021/acs.analchem.7b04099 29183126

